# The next generation of target capture technologies - large DNA fragment enrichment and sequencing determines regional genomic variation of high complexity

**DOI:** 10.1186/s12864-016-2836-6

**Published:** 2016-07-09

**Authors:** Johannes Dapprich, Deborah Ferriola, Kate Mackiewicz, Peter M. Clark, Eric Rappaport, Monica D’Arcy, Ariella Sasson, Xiaowu Gai, Jonathan Schug, Klaus H. Kaestner, Dimitri Monos

**Affiliations:** Generation Biotech, Lawrenceville, NJ 08648 USA; Department of Pathology and Laboratory Medicine, The Children’s Hospital of Philadelphia, Philadelphia, PA 19104 USA; Nucleic Acids & Protein Core Facility, The Children’s Hospital of Philadelphia, Philadelphia, PA 19104 USA; The Center for Biomedical Informatics, The Children’s Hospital of Philadelphia, Philadelphia, PA 19104 USA; Department of Genetics, University of Pennsylvania, Philadelphia, PA 19104 USA; The Department of Pathology and Laboratory Medicine, Perelman School of Medicine, University of Pennsylvania, Philadelphia, PA 19104 USA

**Keywords:** DNA target capture, Targeted enrichment, Genomic resequencing, DNA sequencing, MHC haplotype

## Abstract

**Background:**

The ability to capture and sequence large contiguous DNA fragments represents a significant advancement towards the comprehensive characterization of complex genomic regions. While emerging sequencing platforms are capable of producing several kilobases-long reads, the fragment sizes generated by current DNA target enrichment technologies remain a limiting factor, producing DNA fragments generally shorter than 1 kbp. The DNA enrichment methodology described herein, Region-Specific Extraction (RSE), produces DNA segments in excess of 20 kbp in length. Coupling this enrichment method to appropriate sequencing platforms will significantly enhance the ability to generate complete and accurate sequence characterization of any genomic region without the need for reference-based assembly.

**Results:**

RSE is a long-range DNA target capture methodology that relies on the specific hybridization of short (20-25 base) oligonucleotide primers to selected sequence motifs within the DNA target region. These capture primers are then enzymatically extended on the 3’-end, incorporating biotinylated nucleotides into the DNA. Streptavidin-coated beads are subsequently used to pull-down the original, long DNA template molecules via the newly synthesized, biotinylated DNA that is bound to them. We demonstrate the accuracy, simplicity and utility of the RSE method by capturing and sequencing a 4 Mbp stretch of the major histocompatibility complex (MHC). Our results show an average depth of coverage of 164X for the entire MHC. This depth of coverage contributes significantly to a 99.94 % total coverage of the targeted region and to an accuracy that is over 99.99 %.

**Conclusions:**

RSE represents a cost-effective target enrichment method capable of producing sequencing templates in excess of 20 kbp in length. The utility of our method has been proven to generate superior coverage across the MHC as compared to other commercially available methodologies, with the added advantage of producing longer sequencing templates amenable to DNA sequencing on recently developed platforms. Although our demonstration of the method does not utilize these DNA sequencing platforms directly, our results indicate that the capture of long DNA fragments produce superior coverage of the targeted region.

**Electronic supplementary material:**

The online version of this article (doi:10.1186/s12864-016-2836-6) contains supplementary material, which is available to authorized users.

## Background

Next-Generation Sequencing (NGS) technology has forever transformed the field of genetics, enabling large-scale, high throughput genetic studies for a variety of research and diagnostic applications. While economically sequencing entire genomes remains an important goal of NGS, many research and diagnostic applications are best achieved through targeted DNA sequencing of specific genomic loci. Targeted DNA sequencing is advantageous not only because it is more cost effective, as it facilitates higher sample throughput than whole genome sequencing, but also because it improves accuracy by optimizing the read depth coverage and by reducing the complexity of the DNA to be sequenced.

Several methods have been developed for the targeted enrichment of genomic DNA [1–4] for a variety of clinical and research applications [5–11]. They are typically based upon a multiplexed PCR amplification reaction [12], DNA hybridization to a capture oligonucleotide (either on an array or in solution) [13–15] or DNA capture via molecular inversion probe circularization [16, 17]. Regardless of the method employed, all of these DNA enrichment methods rely heavily on fragmentation of genomic DNA prior to amplification, resulting in relatively short (less than 1000 base-pair) sequencing templates. As a result, existing methods for genomic partitioning remain a severely limiting factor for comprehensively characterizing complex genomic loci because they cannot provide the larger size fragments that are required to successfully span confounding sequence elements, such as extended repeats, or resolve sections of unknown or unexpected sequence that have been inserted or rearranged within the targeted region [18, 19].

Importantly, such large DNA templates can now be utilized by the newer, “third generation” sequencing platforms which are capable of producing significantly larger read lengths [20–22] and sequencing through traditionally difficult sequence templates with high GC content [23]. The longer read lengths produced by these platforms have been shown to be highly advantageous in characterizing structural variants, haplotype phasing within complex genomic loci and *de novo* genome assembly [22, 24–26].

**Fig. 1:**
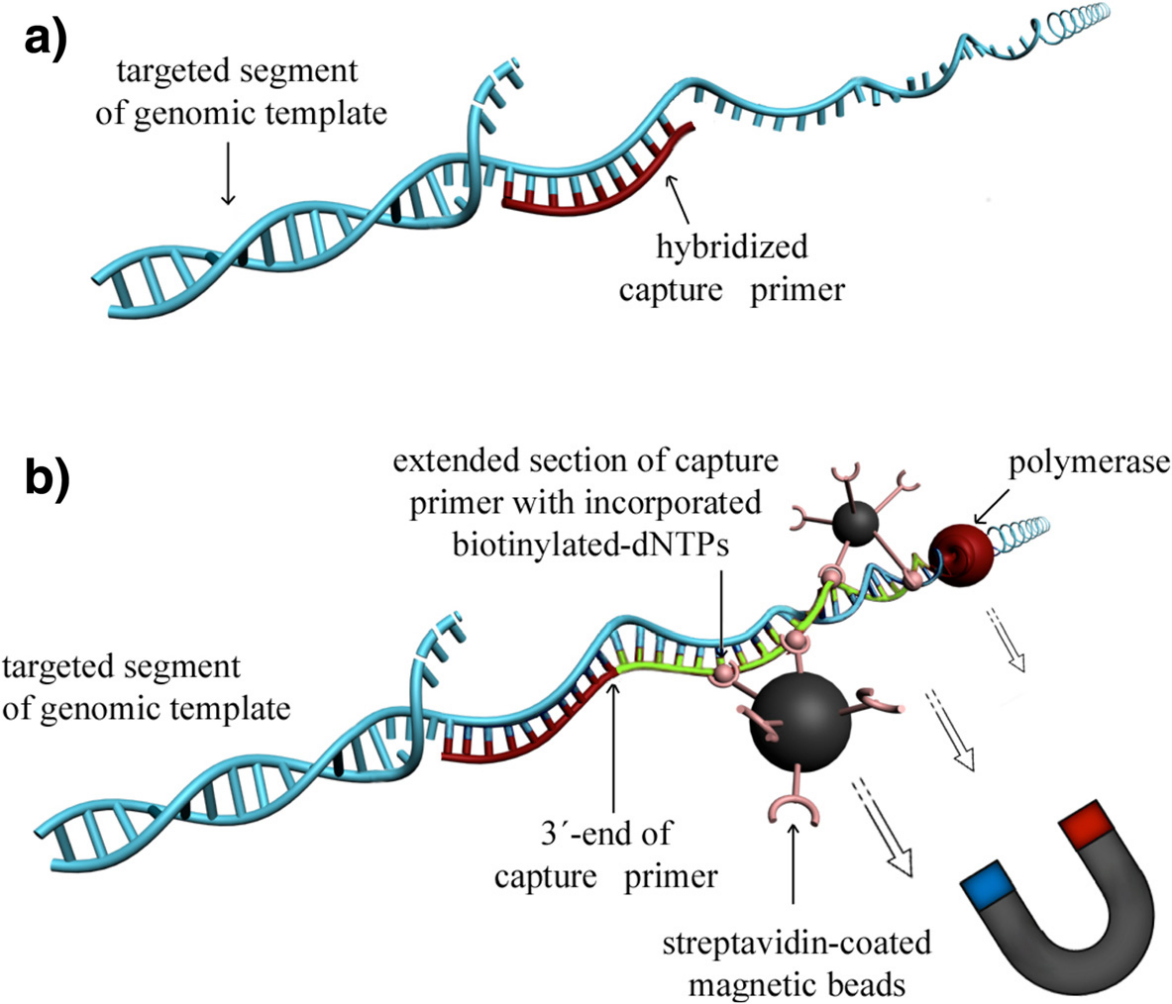
Principle of RSE. **a** During the first step of RSE, the genomic template DNA (*light blue*) briefly gets denatured to allow capture primers (*red*) to hybridize. **b** The bound primers are enzymatically extended with biotinylated nucleotides. The extended portions of the primers, shown in green, form the “handle” to which streptavidin-coated magnetic beads bind. During this process many biotins of the same primer/target DNA complex are bound to streptavidin binding sites on the same bead, thereby forming a topological linkage that firmly locks even very long DNA segments extending in both directions from the capture point onto the surface of the magnetic bead. The primer/target DNA complex is then magnetically purified and released from the bead surface by heat. (The drawing is not to scale: the magnetic beads are approximately an order of magnitude larger than illustrated here)

Our DNA enrichment method, Region Specific Extraction (RSE), addresses this unmet need by capturing long DNA fragments of ≈ 20 kbp in length. RSE utilizes a single primer extension step for capture in which standard oligonucleotides (≈20 bases in length) hybridize to highly specific sequence motifs within the targeted region(s) and are enzymatically extended to include biotinylated nucleotides within the nascent DNA strand. The targeted genomic DNA segments are then pulled down using streptavidin-coated magnetic particles, which bind to the newly synthesized biotinylated DNA sections. These biotinylated portions represent a small percentage of the overall extracted DNA and do not pose a challenge to the efficiency of library preparation and sequencing. The captured segments of the original genomic DNA template, which extend far into both directions from any single point where a capture primer has been hybridized, are then typically amplified by whole genome amplification and processed by standard NGS sequencing protocols (Fig. 1).

A specific program (Antholigo; see “Methods”) we developed for the primer design can be instructed to position the primers at variable distances from their nearest neighbors. If desired, this distance can be 8-10 kbp or greater in order to minimize the number of primers used, while providing for optimal coverage of the targeted region. RSE is simple to use and requires no fragmentation of the genomic sample prior to capture, as other enrichment technologies do. Although the typical size of captured fragments in this study was about 20 kbp, the same principle has been used to extract significantly larger segments depending on DNA quality and the method used for its extraction [27].

Here we demonstrate the utility of RSE for the targeted sequencing of the most complex region of the human genome, the major histocompatibility complex (MHC; HG19 coordinates chr6:29618227-33618227) on the short arm of chromosome 6. The MHC is known to be the most gene-dense region of the human genome, with many transcribed genes playing an important role in innate and adaptive immune processes [28]. Consequently, numerous loci throughout the MHC have been associated with immune-mediated diseases [29–32]. The MHC contains dozens of highly polymorphic genes and large regions of duplication and repetitive elements [28]. Interestingly, despite its significance, there are only two completely characterized MHC haplotypes from two homozygous B cell lines namely PGF (the reference sequence for the MHC in the reference human genome) and COX [33–36]. The same region of the MHC and of the same cell line PGF has been targeted by other capture technologies [15] and offers a unique opportunity for comparisons that demonstrate the advantages of RSE. Eventually this technology can contribute greatly to the comprehensive characterization of such difficult regions around the genome by providing both accurate sequencing and description of structural variations including deletions, insertions and duplications.

## Results and discussion

### Capture efficiency

**Fig. 2:**
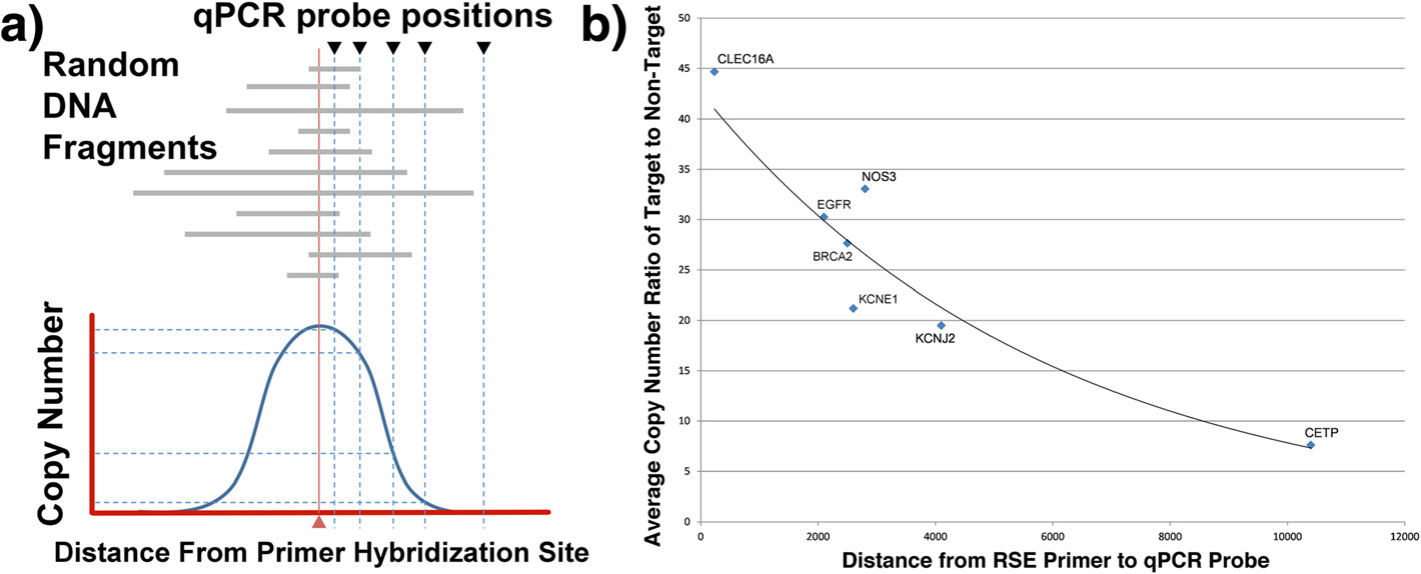
Effects of RSE capture primer spacing on target enrichment. **a** Schematic representation of the distribution of captured genomic DNA copy number obtained around the primer hybridization site, indicated with a red triangle, as measured by qPCRs, placed at increasing distances from the primer hybridization site and shown with black inverted triangles. Gray bars indicate captured random DNA fragments. **b** qPCR results for RSE extracted material at seven non-contiguous genomic regions, plotted as the copy number ratio of targeted sites (indicated as diamonds) to a common non-targeted region (beta actin). The amount of targeted vs. off-target material decreases within about 10 kbp of the RSE extraction site

For a normal distribution of genomic fragment sizes, the highest capture efficiency is observed closest to the RSE primer hybridization site, with decreasing template copy numbers observed further away from the primer hybridization site (see Fig. 2a for schematic representation). We determined the amount of targeted material obtained as a function of distance from the primer hybridization site in order to determine the optimal spacing between designed primers so as to maximize capture efficiency for sequencing and prevent gaps in coverage between adjacent primer hybridization sites, while at the same time, design and synthesize a minimum number of primers.

The optimal spacing of capture primers depends in part on the particular DNA extraction method that was used to prepare the DNA from blood, tissue or cells: The larger the fragments of DNA generated during extraction, the larger the spacing of the primers can be. To study the dependence of capture efficiency at variable distances from the point of primer hybridization, we designed qPCR assays at varying distances from single RSE primers at multiple genomic regions: CLEC16A, EGFR, BRCA2, KCNE1, NOS3, KCNJ2 and CETP (Fig. 2b). We purposely selected unrelated genomic targets to avoid any potential capture bias due to local sequence content and likewise controlled for reliable qPCR amplification at the selected positions. For the DNA used in this study (human genomic DNA extracted by magnetic particle-based isolation on the BioRobot EZ1 (Qiagen), stored frozen in EB and approximately 6-24 months old), qPCR probes located within 1 kbp of a single RSE capture primer generated target to non-target ratios of greater than ≈ 35:1, and primers 2–4 kbp distant produced target to non-target ratios of ≈ 20:1 or greater (Fig. 2b). At a distance of approximately 8-9 kbp from a capture primer, the target to non-target ratio dropped to ≈ 10:1. Estimating this trend beyond 10 kbp, we projected that beyond approximately 25 kbp there will be little to no difference between targeted and non-targeted material for the DNA used here.

### CGH – Determination of effective primer spacing

**Fig. 3:**
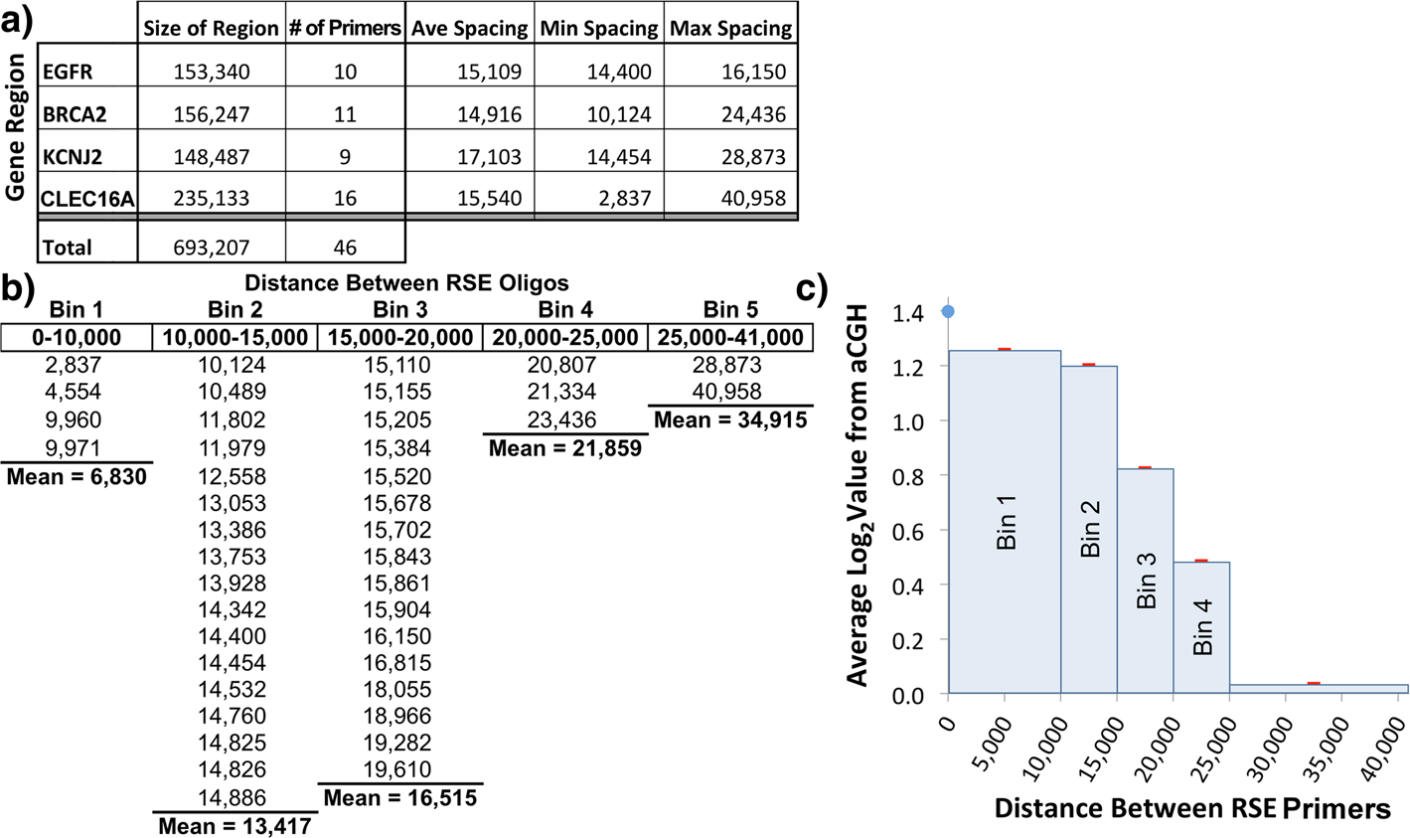
Effects of RSE capture primer spacing on capture effectiveness. **a** 46 RSE primers were designed to capture ≈ 700 kbp of genomic sequence for four gene regions. **b** To examine the effect of RSE primer spacing on capture efficiency, we assumed that the midpoint between the RSE primers would produce the least amount of signal on the array. Each midpoint in the bins shown above was averaged across 20 array primers to account for array probe capture variability. The distance between RSE primers and the averaged array value is presented. **c** The distances between RSE primers were segregated into bins to show the collective effect of similar RSE primer spacing. As seen in the graph, capture of the material as used here at the midpoint between primers drops rapidly beyond ≈ 15 kbp with little to no capture evident at 25 kbp or greater for the type of genomic DNA used in this study (average length ≈ 20 kbp)

To further confirm that the RSE primers are placed at optimal intervals in order to secure continuity of coverage across the targeted region, we utilized a comparative hybridization array. Since some RSE primers are more effective than others (presumably due to regional sequence differences), we investigated the nominal primer spacing necessary to maintain sufficient enrichment for reliable sequencing across a large region (Fig. 3a). Four gene regions (EGFR, BRCA2, KCNJ2 and CLEC16A) that had previously been analyzed on a custom Agilent comparative genomic hybridization (CGH) chip were selected for extraction by RSE. For each region, between 9 and 16 RSE primers were designed with an average spacing of ≈ 16 kbp (Fig. 3b). The captured genomic DNA was then fluorescently labeled with Cy3/Cy5 and hybridized to the arrays.

As seen in Fig. 3c, the hybridization signal (Log_2_Score) at the mid-point between RSE primers decreases rapidly with increasing spacing. As predicted by the qPCR data (Fig. 2b), the array results suggest that primer spacing intervals greater than ≈ 25 kbp produce no net increase in the amount of targeted material retrieved per capture primer. For the purpose of defining sufficient enrichment for sequencing, the results suggest that primer spacing of <10 kbp is required to avoid dropout of target template due to any capture efficiency differences that may occur between primers.

### RSE primer design and MHC capture

The RSE capture method was tested and evaluated on the human MHC because the MHC represents an important and highly complex genomic region that challenges the sequencing enhancement abilities of RSE in a “worst case” scenario. For validation, the homozygous cell line PGF was chosen because a majority of the MHC region within the human reference sequence is based exclusively on the PGF haplotype. A stable and well-characterized reference genome is critical for detecting variant calling errors caused by potential sequence alignment problems, assessing any observed gaps in read coverage and a comparison with existing DNA capture methodologies, which were also evaluated on the MHC [15].

Based upon the qPCR and array CGH results, we designed RSE primers to capture the 4Mbp of the MHC (HG 19 coordinates chr6-29618227-33618227) of the homozygous cell line PGF. The primers were designed based on the reference MHC sequence of the reference genome (HG19). Using in-house developed software (antholigo.chop.edu), 500 RSE primers were designed at ≈ 8 kbp intervals of across the MHC (Additional file 1: Table S1) with an average melting temperature of 58 °C and a target GC content of 50 % (+/-10 %). The primers are designed with similar biophysical characteristics that optimize their performance in the capture reaction, which requires the hybridization of oligonucleotides to genomic DNA.

### Sequencing of the MHC

**Fig. 4:**
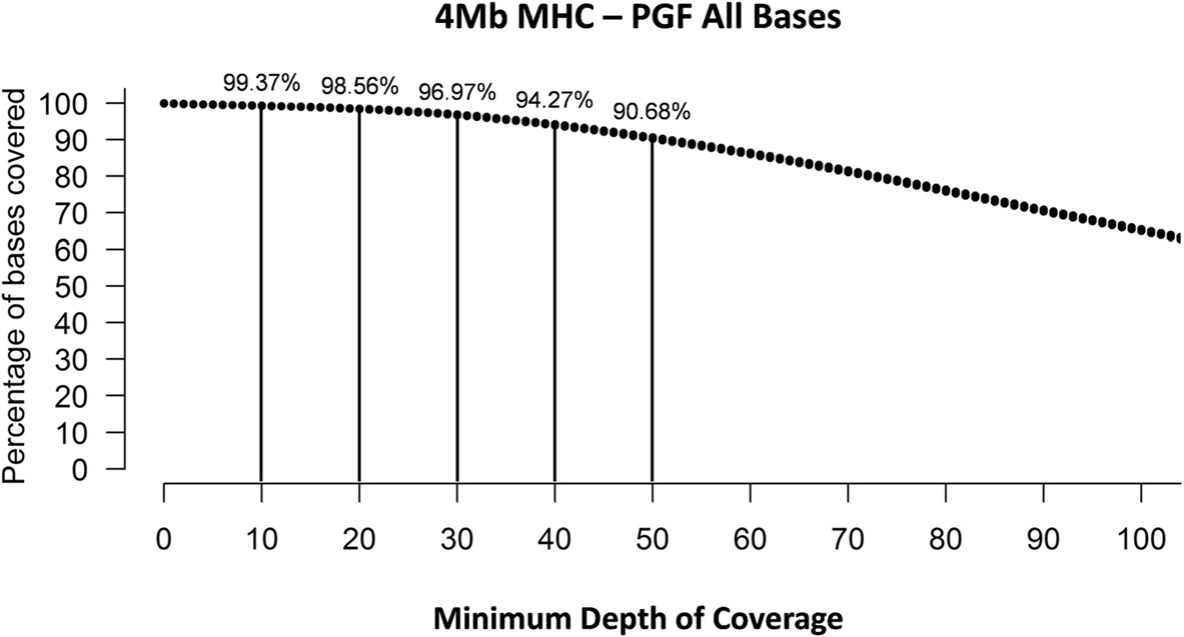
Sequencing depth of coverage of the enriched MHC. The RSE enrichment process results in clinical sequencing depth (>30×) for ≈ 97 % of all enriched bases with >90 % coverage at 50× or greater

The RSE extracted material was then sequenced using 125b paired-end reads on an Illumina GAIIx. Raw data (fastq) files have been made publicly available and are accessible through the NCBI SRA website (SRA accession: SRP075425). Out of 154,822,132 reads, 134,514,112 remained after trimming for quality. Of 67,257,141 reads that mapped to the entire human genome, a total of 6,951,692 reads mapped to the targeted MHC region (Table 1). It therefore derives that about 10 % of the reads were mapped to the MHC, while 90 % were mapped to the rest of the entire genome. The depth of coverage of the targeted MHC region was, on average, very high (164×) compared to the average coverage of non-targeted material (2×) (Table 1). So despite the fact that only 10 % of the reads were mapped to the MHC, the depth of coverage (164×) across the MHC was significantly higher than that across the rest of the genome (2×). Importantly, high depth of coverage was maintained for a majority of the targeted region with 98.56 % of all MHC bases covered at 20× or greater and 90.68 % at 50× coverage or greater, including the known homologous and highly repetitive sections of the MHC (Fig. 4). Since more than half of all bases within the MHC (52.68 %) are repetitive elements, we also evaluated the sequencing results within stretches of unique sequences, which was shown to have coverage depth in excess of 173× (Table 1).

**Table 1:** Sequencing results

Targeted Region (bp)	4,000,002	Targeted Bases Called	3,997,493	Depth >1	99.937 %
Unique Bases (bp)	1,895,669	Unique Bases Called	1,891,678	Depth >1	99.789 %
% of Repeat Sequences	52.68				
% of Unique Sequences	47.39				
Total # of Reads Mapped to Whole Genome					67,257,141
Total # of Mapped Reads to Targeted Region					6,951,692
Average Depth of Coverage for Entire Genome (Non-Targeted)					2
Average Depth of Coverage for Entire Targeted Region					164
Average Depth of Coverage for Unique Sequence in Targeted Region					173

**Fig. 5:**
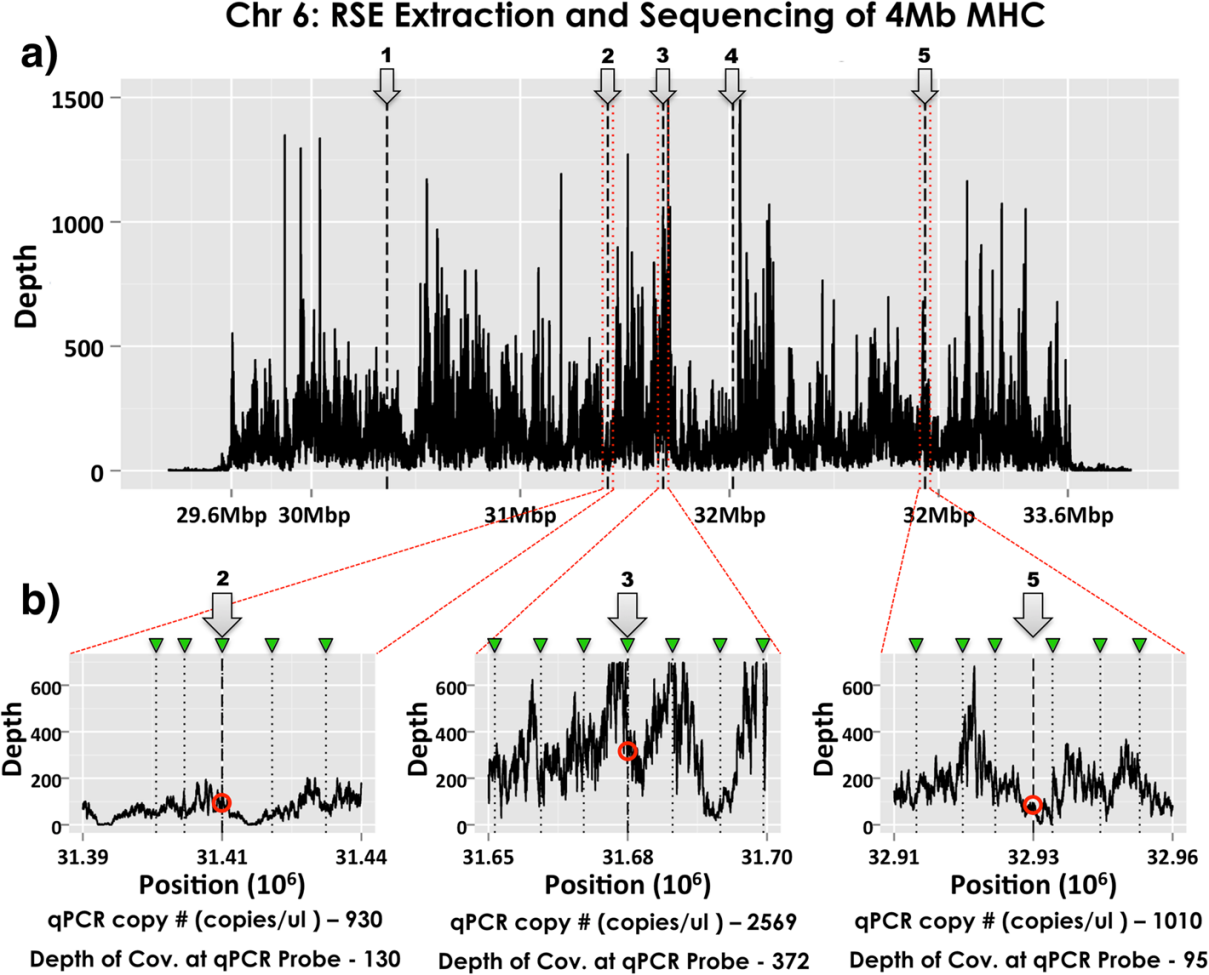
Sequencing depth of coverage map for RSE-extracted MHC region. **a** MHC sequencing coverage is displayed for the entire enriched 4 Mb of the PGF MHC region along with 300 kbp of non-targeted sequence on either side. Each qPCR probe assay is marked by a numbered arrow. **b** 50 kbp regions around each of three qPCR assays is shown to demonstrate differing levels of coverage. RSE capture primer positions are marked with a green marker. The red circle shows the approximate depth of coverage at the qPCR probe position. While regions 2 and 5 have differing average depth of coverage, the qPCR results at the site of capture are very similar (930 vs 1010 copies/μl) which suggest similar amounts of enrichment that is validated by the sequencing depth of coverage results (130 vs 95). Region 3 shows enhanced depth of coverage and suggests higher enrichment that is validated by the higher qPCR results (2569 copies/μl). The depth of coverage results correlate well to the qPCR copy number estimates of the extracted material: higher enrichment = higher depth of coverage

To assess the relationship between the enrichment efficiency as evaluated by qPCR and the final sequencing data after sequencing and assembly, we evaluated the relationship of the absolute copy number obtained from the enrichment process to the sequence coverage. As seen in Fig. 5a, coverage was consistent across the entire MHC region with 99.937 % of the 4 million targeted bases being called. The absolute enrichment was verified by quantitative PCR at five sites (randomly chosen) across the MHC (Fig. 5a). Each qPCR assay site (See Additional file 1: Table S2 for a list of primers and probes) was tested for copy number both, before and after whole genome amplification, of the enriched material and compared to the sequencing coverage obtained directly at the position of the qPCR probe. Figure 5b displays three of the qPCR sites expanded, +/- 25 kbp on either side of the primer to show sequencing coverage at the respective qPCR primer positions.

As seen in Table 2, there is good correlation (0.94/R^2^ ≈ 88 %) between the qPCR results for non-amplified RSE extracted material and whole genome amplified material, with even greater correlation between WGA material and sequencing coverage depth at the qPCR site (0.97/R^2^ ≈ 94 %). This demonstrates that amplification procedures used during NGS library generation do not lead to substantial imbalances between different regions and that sequencing coverage approximates the amount of extracted material obtained through RSE. An overall evaluation of targeted enrichment efficiency based upon sequencing results is an enrichment factor of 82.7 fold for the entire MHC (see in “Methods-Enrichment Determination” for the exact calculations) or in other words the average depth of coverage for the MHC, which was 164×, is 82× more than the rest of the genome, which was 2 × .

**Table 2:** qPCR correlation to sequencing coverage

	qPCR Probe Position within MHC					Corr. Coef	Corr. Coef
	30362055	31417450	31682240	32016911	32935499	1&2	2&3
(1) non-Amped	1565	930	2569	1227	1010	0.94	
(2) WGA	4,201,954	3,312,705	12,750,000	5,974,923	2,337,060		0.97
(3) Coverage Depth	166	130	372	253	95		

(1) & (2) results are copies of target per ul of extracted material

**Fig. 6:**
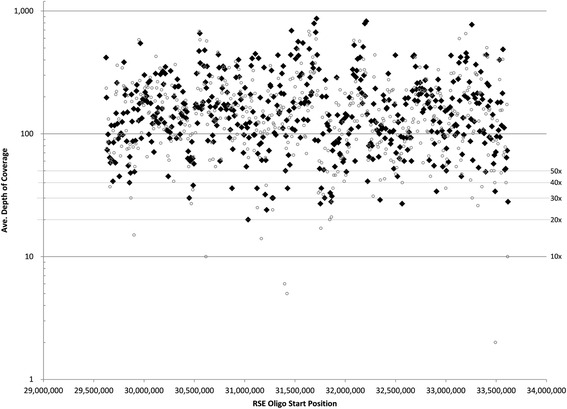
Average depth of coverage at the site of capture and midpoint between capture primers. Average depth of coverage was calculated across all bases underlying each RSE capture primer position. Black diamonds represent the average depth of coverage at the RSE primer position while open circles represent the average depth of coverage at the midpoint between adjacent RSE primers. Out of 500 RSE primers, only 7 were at a depth of coverage of <30× at the RSE capture site (≈ 99 % produced 30× coverage or better) while only 16 midpoints between RSE primers were at a depth of <30× (≈ 97 % of the midpoints were 30× and above)

Evaluating overall RSE primer effectiveness in capturing the MHC, we looked at the depth of coverage at each RSE primer position (Fig. 6). Out of 500 RSE primers, only 7 were found to produce a depth of coverage of less than 30×. This translates into ≈ 99 % of primers performing at our target coverage of 30× or better (30× being considered sufficient for diagnostic applications). Evaluating the midpoint between RSE primers also validated that the RSE primer positioning of approximately 8 kbp between primer sites was sufficient to obtain robust coverage. In this case, only 16 midpoints were at <30× depth of coverage which means that ≈ 97 % of RSE primers were able to provide adequate (≥30×) depth of coverage across the entire MHC region. This indicates that the RSE primer spacing of 8 kbp was successful at delivering the targeted genomic template across the continuum of the MHC. It should also be mentioned that the variation of depth of coverage observed across the 4 Mb of the MHC in Fig. 5a is most likely reflection of the effectiveness of the different primers for capturing their respective regions. This is clearly supported by the variable depth of coverage observed exactly at each of the primer position shown in Fig. 6.

### Gap-free robustness of capture

From an optimization standpoint, this means that only 16 locations would require further RSE primer design or development to provide better depth of coverage at these sites. Considering that only 500 primers are needed to capture the entire MHC, the need to redesign 16 sites out of 500 from a first pass primer design is further evidence of the robustness of the method. From an overall efficiency standpoint, RSE capture produced an enrichment factor of about 82.7; less than that seen with some other technologies. The reasons for this are in part inherent in the capture of very large fragments: cross-hybridization between targeted and non-targeted regions can occur if the targeted genomic segments contain sequences - such as highly repetitive elements - that are also present on other, non-targeted fragments, unless suppressed by blocking such sites during the extraction. Shearing the DNA before capture can reduce the amount of off-target DNA, however this also decreases the available linkage distance. An alternative way to reduce off target material is the use of freshly prepared DNA. Regardless, however, sufficient enrichment for the region of interest is obtained to detect variants accurately.

In a recent publication whereby the MHC (4.9 Mb) of PGF was targeted using a Roche-Nimblegen array [15], the authors acknowledge the limitation of their targeting approach, as well as of most other methods, stating that they were unable to fully cover long, repeated regions and present 100 % coverage of the MHC region. They were able to cover about 94 % of the MHC, as compared to the 99.4 % covered by the RSE at a depth of ≥10×. They recognize that capturing fragments of 5 kbp or even longer will have a beneficial effect in coverage, particularly for regions that are long and highly repetitive.

While current technologies with closely spaced capture oligos suffer from any underlying variability across the targeted area, RSE primers are designed every 8-10 kbp. As such they can be located within regions of lower variability in order to avoid any placement in areas of genomic complexity. This strategy does not completely rule out any instances of variability at a chosen primer hybridization site, but it does provide for the greatest likelihood that any given capture primer is unaffected by every known genetic variance. In addition, because of the very short length of RSE primers compared to that of other types of capture probes (or “baits”), the RSE primers can reliably and redundantly be placed throughout even the most inaccessible regions of highly complex genomes (such as certain plant and animal genomes) that typically get completely repeat-masked and leave no adequately spaced unique sequence regions for the binding of larger capture probes (“baits”). Furthermore the Roche-Nimblegen array, mentioned earlier [15] for the capture of the MHC, needs to include probes that cover 85 % of the total bases of the MHC, which is close to 3.4Mbp, while the 500 primers used for the RSE cover about 0.25 % of the MHC, which is about 10kbp. Therefore there is a substantive lower number of oligos that need to be synthesized for the RSE vs the array capture method by Roche-Nimblegen.

### Universal capture sets for highly variable target regions

Our current experience is that, irrespectively of the complexity of a genomic region, a single standard set of primers can be designed and is able to successfully capture the full extent of complex targeted regions of many different DNA samples (the 4 Mb of the MHC from five other homozygous cell lines have been successfully captured and sequenced; data not shown). This is possible because, in contrast to other capture methodologies, the number of primers to target a particular region is over 100 fold reduced and we can therefore create a near-universal primer set for any given region that contains at least a number of conserved reference points; even when the target region is otherwise highly polymorphic or contains unexpected, unknown sequence or repeat elements.

In the event that some of the designed primers do turn out to be ineffective in terms of capturing a particular region because of local variation, the nature of the method is such that such primers can easily be redesigned and included in the mixture of primers that capture the entire region of interest, without the need to change or re-synthesize any of the others. The capture primers are only about 20-25 nucleotides long, which means that the vast majority of the targeted region never directly comes in contact with any capture primers. This is important because it makes the enrichment nearly immune to pull-down failure and allelic dropout in the case of genomic variations that have not been or could not be anticipated in the capture primer design.

In comparison, other methods may require capture probe/primer sets with a combined coverage of the probe/primer that sums up to more than half the sequence of a targeted region (85 % in the case of Roche-Nimblegen array for the MHC [15]). This leads to a correspondingly high risk of pull-down failure when mutations occur in the region because any stretches of unknown or unexpected sequence within the target region will likely be missed since no capture primers were designed for them. It is important to note at this point that in order to enable streamlined applications in medical diagnostics and theranostics, a single set of reliable capture primers is essential. Designing a single set of reliable capture primers can also be a valuable asset for navigating around other complex and variable sequences that are not fully referenced yet, such as oncology specimens and many plant or animal genomes that are of scientific and economic interest.

### Variant analysis and sanger validation

From a sequencing perspective, the ultimate goal is to provide high depth of coverage across an entire genomic region to promote accurate alignment and variant calling, including sections of the targeted region that may contain unexpected or novel sequence variants. With an average depth of coverage of 164× for all 4 Mb of the targeted MHC, the RSE extraction produces very high depth of coverage. The greatest advantage of RSE is that the depth of coverage is maintained contiguously for almost all bases across the entire region - not merely for exonic regions – and therefore includes important elements such as regulatory and promoter regions that may be key to explaining phenotypes associated with diseases.

The sequencing results were then analyzed to detect variations between the reference genome and the PGF sequencing data. Out of 4 million bases sequenced, a total of 430 variants were found with 409 being single nucleotide substitutions and 21 being in/dels. This defines a variant load of ≈ 1 variant per 9302 bp for the targeted region. Among unique sequences, 102 variants were found corresponding to a variant load of 1 in 18,585 bp. Raw variants were filtered to include only those variants with a coverage depth greater than 20× and a QUAL score greater than 30, leaving 92 identified single nucleotide substitutions and 10 indels.

With 20× depth of coverage, the alignment of the MHC region was sufficiently accurate to detect a surprising number of variations in an otherwise “stable” cell line. In comparing the discovered variants to the reference genome, it was important to discern how many of these variants were reference errors and how many were NGS errors. The NGS data was therefore validated by performing Sanger sequencing on a subset of variants that were located within unique sequences. This analysis included 83 single nucleotide substitutions and 3 in/dels. Table 3 shows the distribution of variant data in light of Sanger validation of the NGS results. Of the 86 variants tested by Sanger sequencing, 61 were located directly within gene regions and included 3 in/dels. Of these variants, 8 were single nucleotide substitutions within exons and 50 were within introns. Twenty-five total variants found were single nucleotide substitutions within intergenic regions. In comparing NGS sequencing results to the Sanger validation sequencing, we found that a total of 50 variants were both detected by the NGS sequencing and confirmed by Sanger sequencing. Thirty-five of these variants are clearly reference sequence errors since the single nucleotide substitutions represent a homozygous (a/a) to homozygous (b/b) allele switch (an unlikely biological scenario in a homozygous cell line). Fifteen of these variants in the PGF cell line grown in our lab were found to be heterozygous (homozygous reference, heterozygous NGS/Sanger sequencing results) which suggests possible *de novo* polymorphic changes. Additionally, 36 variants found by NGS sequencing at more than 30× depth of coverage were not supported by Sanger sequencing (for the complete variant table, see Additional file 1: Table S4). In these cases, the Sanger results supported the reference genome, suggesting that these errors may be attributed to the NGS workflow. Further systematic investigation into the potential cause of these NGS related errors (data not shown) reveals a number of different reasons, including base modifications introduced during WGA or library preparation and Illumina sequencing errors located towards the end of the sequencing reads or within low complexity regions (high GC content, homopolymer stretches, di and tri-nucleotide repeats etc.). Although the sequencing limitations of the Illumina platform, particularly within low complexity regions have been previously described [37, 38], recent improvements and adaptations of NGS protocols have demonstrated promising results in sequencing difficult templates [39] and warrant further investigation within our particular application.

**Table 3:** Sanger validation of identified NGS variants

	Type of variants	Sanger agrees with NGS	Sanger agrees with reference	Total
61 Sanger Validated Variants (Gene Regions)	Exonic	4	4	8
	Intronic	28	22	50
	Insertions/deletions	3		3
25 Sanger Validated Variants (Intergenic)	Mismatches	15	10	25
		50	36	86

### RSE resolves erroneous classification of MHC variants

Two additional variants were caused by read misalignment from a segmental duplication (Additional file 1: Figure S1) (data not included in Table 3). In this case, read alignment identified a “polymorphism” between the two copies of a known segmental duplication. The erroneous classification of this paralogous sequence variant (PSV) as a SNP in the reference sequence was caused by improper segregation of the reads due to an error in the reference sequence located at one of the duplications: The reference error incorrectly identified the central portion of the duplications as being identical, when in reality there was a single base difference between them. The reference error was confirmed by Sanger sequencing and upon editing of the reference sequence, the NGS reads were properly aligned, eliminating the two identified discrepancies (see Additional file 1: Figure S1). A large portion of the NGS errors have known causes, but clearly premature assumptions should not be made when examining variant tables even when the depth of coverage is very high. We strongly suggest that critical variants be further examined at the bam-file level to determine the likelihood of an NGS error.

### Detecting variation within the HLA genes

Considering that the accuracy of the overall unique MHC sequences was 1 variant in every 18,585 bases and that cumulatively the number of bases for the 6 HLA genes is ≈ 38.4 kbp, it would be expected to have no more than 2 variants in the 6 HLA sequences. However, our HLA typing results were found to be concordant with those alleles previously reported by the MHC haplotype project [33] for the homozygous cell line, PGF, with 100 % sequence identity between our consensus sequence for each locus and the reported allele for each locus considered. The exact typing determined by our method was HLA-A*03:01:01:01, B*07:02:01, C*07:02:01:03, DRB1*15:01:01:01, DQB1*06:02:01, DPB1*04:01:01:01.

### Methodological challenges

Due to the nature of its underlying principle, RSE has distinct advantages over current techniques as well as restrictions that can limit its effectiveness depending on the application. For example, the ability to capture DNA segments that are at least an order of magnitude larger than those isolated with other methods comes with a lower overall capture efficiency per locus compared to short-fragment pull-down methods. For most RSE captures, the maximum amount of material extracted at any targeted region is generally less than 30 % and usually around 10 % of the total DNA captured (Table 1: reads mapped to targeted region/reads mapped to whole genome = 10.34 %), again dependent on the length of the DNA template before RSE and the target DNA’s degree of entanglement with other strands during isolation by RSE.

Hybridization of primers to non-targeted sites and the addition of the capture moiety, biotin, via primer extension-based nucleotide addition can result in random accumulation of off-target material. DNA quality - i.e. primarily age, the method used for its extraction from blood/tissue/cells, and conditions for its storage and handling - play a crucial role here and can be a major cause for off-target material accumulation. Extensive heating also creates DNA damage. For certain applications it may therefore be advisable to perform an alkaline denaturation instead of one by heat.

Lastly, due to the large DNA segments captured by RSE, it also isolates any repeat regions that are located within 10-20 kbp of a target site. If present in high copy numbers, DNA fragments deriving from these segments can result in unintended self-priming events during any required subsequent whole genome amplification (WGA) step, which may disproportionately increase the amount of these sequences if the amplification time is allowed to be long. While there is no indication in the depth of coverage data that this was a significant problem adversely affecting our sequencing results, it is known that excessive WGA times can lead to bias in template over-representation. We therefore recommend limiting any WGA to 1-2 h (for the Qiagen REPLI-g Mini kit) unless forced to work with very small amounts of input DNA for RSE. Recent advances in NGS library preparation protocols have drastically reduced the amount of input DNA needed (< 20-50 ng), which obviates the need to perform the WGA step at all provided that sufficient amounts of genomic DNA template are available for RSE.

The main advantage of RSE is its ability to specifically capture and provide unambiguous sequence data even for DNA sections that are embedded in highly repetitive, complex or unknown regions. The corresponding amount of off-target material still does present challenges to the sequence alignment process and limits the degree of sample multiplexing per NGS run, but it can be controlled to some degree through the steps listed above. As seen in the sequencing results shown here, the number of reads that aligned to the targeted MHC region is ≈ 10 % of the total number of reads that map to the genome (Table 1). The amount of off-target material is largely spread evenly across the entire genome with very low coverage. This reduces its impact and allows for high quality sequencing and accurate variant detection for large, contiguous genomic regions of interest.

## Conclusions

Region specific extraction is a genomic targeting method with distinct advantages over other, currently employed targeting approaches. By capturing very long (≈ 20 kbp in this study), overlapping DNA segments directly from a sample of genomic DNA, RSE can isolate an entire contiguous target region, including exonic, intronic and intergenic regions. As such it provides the framework for the reliable characterization of any genomic region, regardless of its complexity and variability. With an appropriate sequencing technology that can characterize long fragments (i.e. Pacific BioSciences, Oxford Nanopore), RSE can also provide haplotype phase information, thereby setting the stage for accurate *de novo* assembly of targeted genomic regions such as the MHC.

RSE was able to deliver adequate coverage (99.937 %) and impressive accuracy (99.99 %-1 variant in every 9302 bases) throughout a highly complex target region. While our analysis of short Illumina reads, derived from the homozygous cell line PGF, has facilitated the comprehensive characterization of the PGF MHC through reference-guided read mapping to the corresponding MHC haplotype, RSE is uniquely positioned to characterizing the inherent sequence complexity of the MHC derived from a heterozygous sample. This could be accomplished via sequencing platforms that generate long reads which can be used to phase long stretches of repeat sequence elements and structural variations present throughout the MHC of a heterozygous sample. Towards this end, our lab has demonstrated the utility of RSE to generate long read sequencing templates from the targeted MHC capture of the homozygous cell lines PGF and COX, and subsequent PacBio sequencing. Our initial results produce PacBio sequencing reads with an average length of ≈ 4.5 kbp and very encouraging *de novo* assembly results (manuscript in preparation), suggesting that haplotype resolved *de novo* assembly of heterozygous MHC samples is feasible.

## Methods

### Genomic DNA preparation

The PGF and GM 12248 (CEPH collection) cell lines were obtained from Coriell Cell Repositories. Cells were cultured overnight at 37 °C, 5 % CO_2_. On the next day, cells were split into two 75 cm^2^ culture flasks and cultured in 10 ml of RPMI1640 containing 10 % Fetal Calf Serum at 37 °C, 5 % CO_2_. After obtaining 2 × 10^7^ cells per flask, gDNA was extracted using a Blood & Cell Culture DNA Midi Kit (Qiagen, Cat# 13343).

### Whole genome amplification

The enriched samples were amplified with REPLI-g Midi Kit (Qiagen Cat.# 150043) according to manufacturer’s protocol using 5 μl of RSE template and incubating at 30 °C for 16 h followed by inactivation of the enzyme at 65 °C for 3 min. Residual primers and dNTPs were deactivated with ExoSAP-IT (Affymetrix, Cat.# 78201 1 μl) according to the manufacturer’s protocol (http://media.affymetrix.com/support/technical/usb/brochure/ExoSAP-IT_Brochure.pdf).

### RSE capture primer design

The design of RSE primers used for the capture of targeted regions was performed using a custom designed software program called RSE Antholigo (available via http://antholigo.chop.edu/dgdweb/oligo/home.html). It utilizes and integrates tools and databases to automatically generate primer sets that satisfy several user-defined criteria at predefined genomic intervals. It accesses a local installation of the UCSC database and human genome sequence library downloaded from UCSC Genome Browser to retrieve DNA sequences that are masked for repeat regions and SNPs. A genomic region of interest is parsed into smaller regions in which the primers are designed approximately equidistant to each other based upon user settings (currently, the primers are 6-10 kbp apart). It then uses the primer design software Primer3 [40] to design the primers based on user-defined criteria including GC content, melting temperature and length. After primers are chosen, homology between selected primers and the rest of the genome is checked with BLAT [41].

The program targets conserved sequence across the region of interest. The RSE capture primers had a melting temperature of approximately 58 °C and GC content of 50 % (+/-10). Antholigo uses proprietary software UNAFold [42] that performs a pair-wise assessment of all primers to ensure minimal primer dimer formation and minimal hairpin formation. In this experiment, 500 capture primers were designed to target the entire 4 Mbp of the MHC (HG19 coordinates chr6:29618227-33618227) at an average spacing of ≈ 8 kbp and a target to primer sequence coverage ratio of > 300 (see Additional file 1: Table S1 for the list of primers and supporting information). The primers were synthesized by IDT (Integrated DNA Technologies), (Coralville, IA) and provided in their “Lab Ready” format, pre-diluted to 100 μM. Primers were then combined (in water) to an equimolar ratio of all 500 primers.

### Region Specific Extraction (RSE)

Each 30 μl RSE reaction contained approximately 550 ng genomic DNA, 5 μM region specific primer mixture, H-Buffer containing polymerase, dNTPs and biotinylated dNTPs (Generation Biotech, Prod.# 720; www.generationbiotech.com) and DNAse-free water. Extractions were placed on a heat block with a heated lid (SciGene Hybex™; www.scigene.com/details.php?pid=1180) to denature the DNA at 95 °C for 7.5 min. The samples were then transferred to a BioRobot EZ1 (Qiagen). An automated protocol completes a 20 min incubation at 64 °C during which the region specific primers anneal and are enzymatically extended, incorporating a mix of unmodified and biotinylated dNTPs. The targeted genomic DNA was captured by incubating with 60 μl of streptavidin-coated magnetic microparticles (Generation Biotech, Prod.# 710) at room temperature on the EZ1 following GB protocol. The EZ1 protocol washes the microparticles containing the captured DNA to remove non-targeted DNA. The particles carrying the targeted DNA are then collected and resuspended in 50 μl Qiagen EB buffer on the EZ1. The captured DNA is then removed from the magnetic particles by heating the solution at 80 °C for 15 min and magnetically collecting the particles. The target DNA is retained in the supernatant.

### Capture efficiency assessment

The capture efficiency at different distances from the primer hybridization site was assessed using, seven loci (CLEC16A, EGFR, BRCA2, KCNE1, NOS3, KCNJ2 and CETP) all outside the MHC, by qPCR (quantitative PCR) using RSE captured material from the GM 12248 DNA sample. For each 25 μl reaction, 10 μl of enriched DNA sample were combined with 1× Qiagen Quantitect Probe PCR master mix (Cat. # 204345), 0.4 μM each of forward and reverse primers (IDT) and 0.2 μM probe (IDT). See Additional file 1: Table S2 for a list of qPCR primers and probes.

For the five qPCR assays used to quantify the PGF MHC extraction (Additional file 1: Table S2, MHC-1, MHC-2, MHC-3, MHC-4, MHC-5), 10 μl of sample were combined with 1× Qiagen Quantitect Probe PCR master mix (Cat. # 204345), 0.4 μM each of forward and reverse primers (IDT) and 0.2 μM probe (IDT) for assays in target region. Six 1:3 serially diluted GM12248 or PGF genomic DNA standards were run in duplicate for each locus as well as a single negative control. Forty cycles of (95 °C for 15 s, 60 °C for 1 min) were run after the initial denaturation at 95 °C for 15 min. Fluorescence was collected at 60 °C. The selection of sites for the design of the qPCR assays is made accounting for the sequencing characteristics of a particular region and its surrounding context, such that each assay will have reasonable chances to work. Depending on the size of the targeted region, multiple qPCR assays should be designed for both target and non-targeted regions for an accurate estimation of enrichment and of the corresponding depth of coverage that can be achieved after sequencing (See Additional file 1: Table S2 for a list of qPCR primers and probes).

### Agilent custom array comparative genomic hybridization

Comparative Genomic Hybridization was used to assess optimal spacing of primers. Four loci (EGFR, BRCA2, KCNJ2 and CLEC2) were targeted with 46 primers (See Additional file 1: Table S5 for the complete list of primers used to capture the four regions) using genomic DNA from GM 12248 cell line. DNA was prepared using standard automated bead-based methods from Qiagen. This process generally produces genomic fragments in the 20–40 kbp range. A custom Agilent 8 × 15 K Agilent Comparative Genome Hybridization microarray (CGH) was designed using Agilent’s online tools (https://earray.chem.agilent.com/suredesign) and ordered using their standard custom array process. The array was used according to the manufacturer’s protocol, ver 5.0. Briefly, genomic DNA and whole genome amplified RSE extracted material was restriction digested with Alu I and Rsa I. The digested material was then labeled with Cy3 or Cy5 using the random priming process of the manufacturer. Labeled samples were then washed, filtered and checked for expected yields. 8 μl of each labeled sample was then prepared for hybridization by placing in blocking buffer (Cot-1, Agilent 10× blocking buffer and 2× hyb buffer), heated to 95 °C for 3 min, then placed in a water bath at 37 °C for 30 min. The custom array was prepared as per instructions. Sample was hybridized to the array at 65 °C for 24 h. The hyb cassette was opened and the array washed per instructions. The array was scanned at 5 μm resolution. Raw data was analyzed with the Agilent Feature Extraction software version 9.5 using default parameters.

### Enrichment determination

Targeted enrichment values were calculated from Illumina sequencing read data using the formula from Gupta, et al. [43]:

$$ \frac{\frac{\left( number\kern0.5em  of\kern0.5em  reads\kern0.5em  that\kern0.5em  map\kern0.5em to\kern0.5em  the\kern0.5em  target\kern0.5em  region\right)}{\left( total\kern0.5em  number\kern0.5em  of\kern0.5em  reads\right)}}{\frac{\left( target\kern0.5em  region\kern0.5em  size\right)}{\left( haploid\kern0.5em  genome\kern0.5em  size\right)}} $$

Enrichment was estimated based on a haploid genome size of 3.2 Gb for the cell line used and on the data shown in Table 1. The enrichment was: (6,951,692/67,257,141)/(4,000,000/3.2*10^9) = 82.7.

### Illumina GAIIx sequencing

Five micrograms of enriched, amplified material were used as input for preparation of the sequencing library. The library was prepared for sequencing using the Illumina Paired-End DNA Sample Prep Kit (Cat. # PE-102-1001), according to the manufacturer’s protocol. Sequencing was performed using an Illumina Genome Analyzer IIx, 2 × 125 base paired-end chemistry. Raw data (fastq) files have been made publicly available and are accessible on the NCBI SRA website (SRA accession: SRP075425).

### Sanger sequencing

To validate variant calls, genomic or whole genome amplified DNA was used. PCR primers for each variant were designed manually using the IDT website (www.idtdna.com). The full list of PCR primers is provided in Additional file 1: Table S3.

To set up each PCR reaction, 150 ng of gDNA or whole genome amplified DNA was combined with 10× PCR buffer (Qiagen), 10 mM dNTP mix (Life Technologies, Cat. # 18427088), 5U/μl HotStar Taq DNA Polymerase (Qiagen, Cat. # 203203), 5 μM each of forward and reverse primers and water. The thermocycling protocol was 10 min at 94 °C for the initial denaturation followed by 37 cycles of 30 s at 94 °C, 30 s at 53 °C or 57 °C, 30 s at 72 °C and a final extension for 5 min at 72 °C.^1^ After PCR, 2 % gel electrophoresis was performed to validate amplification. Then each amplicon was purified with 4 μl of ExoSAP-IT (Affymetrix, Cat. # 78201 1 μl) by incubation for 45 min at 37 °C, followed by inactivation of the enzyme for 15 min at 80 °C. Sanger sequencing was performed using the same primers, which were used for PCR amplification. Two μl of each amplicon were combined with 0.5 μl of 3.2 μM of each forward or reverse sequencing primer, 1.5 μl Big Dye 5× sequencing buffer, 1 μl Big Dye Terminator v 1.1 (Life Technologies, cat#4336701) and 5 μl water. The cycle sequencing protocol was 10 s at 96 °C followed by 25 cycles of 10 s at 96 °C, 10 s at 50 °C and 2 min at 60 °C. Reactions were precipitated with 2 μl NaOAc/EDTA buffer, followed by a wash using absolute ethanol and a second wash using 80 % ethanol and resuspended in 15 μl HiDi-formamide. The raw data was analyzed with Sequencing Analysis software, version 5.2 (Life Technologies/Applied Biosystems).

### Sequence alignment & variant detection

PGF sequencing data was generated from 125 bp paired-end reads generated on the Illumina GAIIx sequencing platform. The paired-end reads were quality trimmed (minimum PHRED score of 30) using sickle version 1.010 (https://github.com/najoshi/sickle). Quality trimmed reads were aligned to the reference genome (HG19) using BWA version 0.6.2-r126 with default parameters [44]. After alignment, variant calling was performed following GATK v3 best practices with recommended parameters for accurate and efficient variant calls [45] (GATK version 1.6-2-gc2b74ec and Picard version 1.57). The only step not applied was the removal of duplicates since the target (4 Mb of the MHC) was relatively small compared to the whole human genome.

### Sequence variation within HLA loci

Reads mapped to the reference genome (HG19) were used to generate a consensus sequence for all HLA loci considered for typing (HLA-A, HLA-B, HLA-C, HLA-DRB1, HLA-DQB1, HLA-DPB1). For each locus, a consensus sequence was generated from the set of mapped reads using Samtools [46]. The consensus sequence obtained from each locus was then aligned pairwise against each fully characterized allele within the IMGT database (ImMunoGeneTics; www.ebi.ac.uk/ipd/imgt/hla) for each respective locus using the Needleman-Wunsch algorithm as implemented within MATLAB. The highest alignment score was then used to call the allele for each locus and the percent sequence identity between a given consensus sequence and assigned allele for the particular locus in question was also calculated.

## Abbreviations

CGH, competitive genomic hybridization; MHC, major histocompatibility complex; NGS, next generation sequencing; PSV, paralogous sequence variant; RSE, region specific extraction; WGA, whole genome amplification

## Additional file

Additional file 1: Table S1. 500 RSE capture primers designed using Antholigo. The genomic position (HG19) of the primers as well as the sequence and spacing between primers is provided in addition to other design criteria metrics including deltaG, Tm, and GC%. **Table S2**: List of qPCR primers and probe sequences designed to amplify regions within several genes (ACTB, EGFR, CLEC16A, BRCA2, CETP, KCNJ2, KCNE1, NOS3) as well as five MHC locations (MHC-1, MHC-2, MHC-3, MHC-4 and MHC-5 as indicated by arrows in Fig. 5a). **Table S3**: PCR primer location and sequences designed to validate high-confidence variant calls from NGS results. **Table S4**: Results from Sanger validation of high confidence variants identified by NGS. **Table S5**: 46 RSE primers designed to capture the four genes (~700 kb in total) in order to examine the effect of RSE primer spacing on capture efficiency (Fig. 3). Figure S1: Read segregation problems caused by reference sequence error in regional duplication. A 2.5 kb duplication (a) within the MHC presented difficulties during the alignment of reads to this region. Reads at the beginning and ends of the duplicated regions segregated cleanly due to polymorphic differences between the duplications. But reads aligning to the middle of the duplication identified a variant position (marked by an *) (b). Sanger sequencing of this region identified that the reference sequence incorrectly identified the * base in the second duplication as a T, when it was actually a C, creating a false variant position. After correcting the reference sequence (c), the central reads segregated correctly and the false variant was eliminated. (PDF 1372 kb)

## Footnotes

1 Different temperatures were used depending on Tm of the primer pair (see Additional file 1)
