# Supplementary material for: The next generation of target capture technologies - large DNA fragment enrichment and sequencing determines regional genomic variation of high complexity
Source: BMC Genomics. 2016 Jul 9;17:486. doi: 10.1186/s12864-016-2836-6 (PMC4938946; doi:10.1186/s12864-016-2836-6)
Supplement: Additional file 1: Table S1. — 500 RSE capture primers designed using Antholigo. The genomic position (HG19) of the primers as well as the sequence and spacing between primers is provided in addition to other design criteria metrics including deltaG, Tm, and GC%. Table S2: List of qPCR primers and probe sequences designed to amplify regions within several genes (ACTB, EGFR, CLEC16A, BRCA2, CETP, KCNJ2, KCNE1, NOS3) as well as five MHC locations (MHC-1, MHC-2, MHC-3, MHC-4 and MHC-5 as indicated by arrows in Fig. 5a). Table S3: PCR primer location and sequences designed to validate high-confidence variant calls from NGS results. Table S4: Results from Sanger validation of high confidence variants identified by NGS. Table S5: 46 RSE primers designed to capture the four genes (~700 kb in total) in order to examine the effect of RSE primer spacing on capture efficiency (Fig. 3). Figure S1: Read segregation problems caused by reference sequence error in regional duplication. A 2.5 kb duplication (a) within the MHC presented difficulties during the alignment of reads to this region. Reads at the beginning and ends of the duplicated regions segregated cleanly due to polymorphic differences between the duplications. But reads aligning to the middle of the duplication identified a variant position (marked by an *) (b). Sanger sequencing of this region identified that the reference sequence incorrectly identified the * base in the second duplication as a T, when it was actually a C, creating a false variant position. After correcting the reference sequence (c), the central reads segregated correctly and the false variant was eliminated. (PDF 1372 kb) [file 12864_2016_2836_MOESM1_ESM.pdf]

Primer ID	Start	End	Distance	Sequence	GC%	TM	Size	Self dG	Hairpin Tm	Hairpin dG
MHC 1b 29730938	29730938	29730963	4136	TGAGGCAGTTGAGGGCATGAAAGTA	48	58	25	-5.4	30.4	-0.43
MHC_2_29735074	29735074	29735099	7493	GGGCAGTTCAGAGTGATAGGACCAA	52.0	58.0	25	-3.5	16.6	0.61
MHC_3_29742567	29742567	29742592	7705	GCCTTCTCCTCCTACCAAGTGTCTC	56.0	58.1	25	-3.5	17.5	0.36
MHC_4_29750272	29750272	29750297	9109	CCCATAGACAATCCACAGACACCCA	52.0	58.0	25	-1.6	0.8	0.96
MHC_5_29759382	29759382	29759406	7498	CGCAGAAAATTACAGAGACGCACA	45.8	56.3	24	-5.4	19.6	0.39
MHC_6_29766876	29766876	29766904	6617	TGTGCTGTATAGTCTTCCTATTTGAGAA	35.7	54.0	28	-4.0	32.3	-0.67
MHC_7_29773495	29773495	29773521	8990	ACAAGCAATACTCTCTGATTTGAAGG	38.5	53.7	26	-3.9	27.2	-0.2
MHC_8r_29782486	29782486	29782511	8579	CCC AAT CCT CTT TCC TGC TGT TGA T	48.0	57.0	25	-3.9	18.4	0.57
MHC_9_29791065	29791065	29791090	8085	TCCCGTTTCTCTCCACCTATTCTCT	48.0	56.5	25	-3.6	-6	1.86
MHC_10_29799150	29799150	29799175	5705	AGTTCCCAGGTTCTAAAGTCCCACG	52.0	58.2	25	-3.6	20.6	0.32
MHC_11r_29804855	29804855	29804880	9503	TGCTGGCTCACAATGGCTTATGTTT	44.0	57.7	25	-3.3	23.7	0.09
MHC_12_29814358	29814358	29814383	8964	CTTCAGTCAGGACACAGGACAGAGT	52.0	57.7	25	-2.9	34.5	-0.86
MHC_13_29823322	29823322	29823347	7741	CTTTGGTTAGGGTGAGGACAGGAGG	56.0	58.2	25	-2.0	22	0.16
MHC_14r_29831063	29831063	29831088	7567	TCTCAACCCAAAGACACCATGACGG	52.0	58.9	25	-5.4	34.2	-0.5
MHC_15_29838630	29838630	29838655	5718	CAACTGGAGAAGCTGTTAGGTCCCA	52.0	58.2	25	-6.3	23.8	0.07
MHC_16_29844349	29844349	29844373	7037	GTCACTGGCTATGTGTGAGAGTCTC	58.5	52	25	-4.8	37.1	-1.06
MHC_17_29851390	29851390	29851410	11241	CAACACCCAAGGCTCTGTGA	55.0	54.3	20	-3.3	38.5	-1.21
MHC_18_29862627	29862627	29862651	7754	TTCTCCTAAGCACCCAAGGCTCTG	54.2	58.2	24	-4.7	31.6	-0.44
MHC_19_29870379	29870379	29870405	8626	CTTGCTGTTTCCAACTCTTTCCTCCT	46.2	57.3	26	-3.9	28.9	-0.32
MHC_20_29879006	29879006	29879031	11626	ACCACATACTTGAGACCCAGGACAC	52.0	57.9	25	-3.1	29.7	-0.31
MHC_21b_29890632	29890632	29890657	4427	AATCAGCACGCAATTCCCAACTCAC	48	58	25	-5.4	-49.5	1.91
MHC_22r_29895059	29895059	29895084	7713	TGTTACGGTGCCATATACTCTTTGT						
MHC_23_29902772	29902772	29902797	6374	ACACGGAAACTTAGGGCTACGGAAT	48.0	58.0	25	-3.6	28.7	-0.28
MHC_24b_29909146	29909146	29909171	11250	ACA GGG GTG GTG GAA GTA GGA TAG T	52	58	25	-2	10.7	0.91
MHC_25_29920396	29920396	29920421	6569	TTCTCTGATGTCACTCCTCAGCCAC	52.0	58.0	25	-5.1	31.8	-0.71
MHC_26r_29926965	29926965	29926990	8514	AGGCTCACAGGACAACCAGTAAAGA	48.0	57.6	25	-3.1	30	-0.26
MHC_27r_29935477	29935477	29935504	7853	GCA ACT TCT TCT GAT GCT TTA TGG GCT	44.4	58.0	27	-5.1	32.1	-0.55
MHC_28_29943334	29943334	29943357	7392	CACCCACACAGCAGAGAAGATGC	56.5	57.9	23	-5.1	33.7	-0.68
MHC_29_29950724	29950724	29950749	12284	ATGTGATTGGGCTGTAAGTAAGCAT	40.0	54.8	25	-4.7	38.8	-1.3
MHC_30_29963009	29963009	29963033	3494	AGCTCACTCTCTGTCACCAAACTCC	52	58.2	25	-6.3	10.2	0.63
MHC_31_29966502	29966502	29966527	6821	GCACCTGAATGTGTCTGTGTTCCTG	52.0	58.3	25	-3.5	33.5	-0.76
MHC_32_29973323	29973323	29973348	9400	AAAGGAGTCAGCTATGCTCAGGCTT	48.0	58.0	25	-6.3	38.3	-1.08
MHC_33r_29982723	29982723	29982748	6622	AGGTGCCCATGACAGGATTGAAGAC	52.0	58.7	25	-5.4	27.4	-0.32
MHC_34_29989346	29989346	29989370	7038	CGAAAGGGCAGATACCGTGGAATG	54.2	57.9	24	-3.6	16.9	0.5
MHC_35_29996383	29996383	29996408	8869	ACCCAAGGCTCTGTCATGTCTCAAA	48.0	58.1	25	-5.4	26.1	-0.09
MHC_36_30005253	30005253	30005277	7186	ACCTGAATGTGCCTGTGTTCCTGTA	48	58	25	-3.5	19.6	0.49
MHC_37r_30012438	30012438	30012463	9609	CTGCTACCCAGTCATCCTCCAGAAG	56.0	58.1	25	-3.6	33	-0.59
MHC_38_30022048	30022048	30022072	9886	TACTCTTCTTCCTGCTCTGAGTTGT	44	57.2	25	-4.5	34.8	-1.13
MHC_39r_30031933	30031933	30031958	4817	ATCTTATAGCCCTGAGTTTGTGTGCG	46	57	26	-3.6	11.6	0.91
MHC_40_30036750	30036750	30036775	9650	AGAGGTATTGGCGTATTATCAGAGT	40.0	53.2	25	-3.6	8.5	1.21
MHC_41_30046400	30046400	30046425	7824	TGGAATGGCTGGTCATCAACTCTGA	48.0	57.9	25	-3.5	23.4	0.11
MHC_42_30054224	30054224	30054249	8487	GCCACACACCCAAATAGTTACAGGA	48.0	57.0	25	-3.1	26.6	-0.08
MHC_43r_30062226	30062711	30062736	8389	CTG GAT TGC CCT GTA ACC CCA CAT T	48.0	57.3	25	-5.4	23.5	0.13
MHC_44_30071100	30071100	30071125	9956	GCCTAGAAGACACCAAGAGTTCGCT	52.0	58.5	25	-4.2	10.1	0.58
MHC_45_30081057	30081057	30081081	5177	TGAGTGATAAGAGGGACGGAGGGAA	52	58	25	-3.6	-3.9	1.77
MHC_46_30086232	30086232	30086258	8536	GTTGGAAAATTGGACCCACAAGGCTA	46.2	57.8	26	-5.4	34	-0.5
MHC_47_30094770	30094769	30094794	4909	GTGTACTCCCAGCCCACAATCCTAT	52.0	57.8	25	-3.6	13.8	0.66
MHC_48r_30099679	30099679	30099703	11849	GGCTCCCTCCATGTATCTCCAGTAC						

MHC_49_30111527	30111527	30111552	6905	ATCTCCTGGTTATCTGCTTTCATGG	44.0	54.7	25	-5.4	3.4	1.1
MHC_50r_30118432	30118432	30118457	11071	TTCTCACAAATGCTGTTCTCGCCTC	48.0	57.8	25	-3.6	29.2	-0.25
MHC_51r_30129503	30129503	30129528	4915	CTG AGT TTG GAA ATG GCA GAG TGG G	52.0	58.0	25	-3.9	18.8	0.48
MHC_52_30134418	30134418	30134443	7791	CCCTCTGCTTGCCTGACTAACAATG	52.0	57.9	25	-3.9	16.4	0.52
MHC_53_30142209	30142209	30142234	8196	TGCTTCATCCTACTCATCACCCCAG	52.0	57.8	25	-3.1	-6.1	1.72
MHC_54_30150405	30150405	30150430	5213	CCCTTCCTCCATCCTCTTGTCAGTC	56.0	58.1	25	-2.0	10.5	0.93
MHC_55r_30155619	30155619	30155643	10736	TCT TTG GGA AGG GCA TAG TGA GTG G	52	58	25	-3.5	33.7	-0.67
MHC_56_30166355	30166355	30166379	8045	CCCATTGCACTCTTGCCTATTCCAC	52	58	25	-7	41.3	-1.4
MHC_57_30174399	30174399	30174424	8064	TATGTTCTCGCTTTCCTGTGGGTGA	48.0	57.9	25	-3.6	30.4	-0.37
MHC_58_30182463	30182463	30182488	8146	AGTCTCCCCAACTCCAAACCTGATG	52.0	58.2	25	-2.9	19.5	0.34
MHC_59_30190610	30190610	30190634	8722	GGCAACCCAGCAGAGAGAATGAAA	50.0	57.4	24	-3.1	36	-0.8
MHC_60r_30199332	30199332	30199356	7418	AGGCTTATCTCTCATTCCCACCCAC						
MHC_61_30206749	30206749	30206774	8005	ACACAGTTTTATGCTCCCCTTCCCT	48.0	57.8	25	-3.1	13.7	0.72
MHC_62_30214754	30214754	30214779	8320	GCCGATGAAGCAGCATAAGATGGAG	52.0	58.1	25	-3.6	29	-0.3
MHC_63_30223074	30223074	30223099	7677	TGGTGACTTGTGGTAGATGTGGCTT	48.0	58.1	25	-3.1	25	0.04
MHC_64r_30230751	30230751	30230776	7463	ACTGAGGCAGAGTGAATAACCCAGG						
MHC_65c_30238214	30238214	30238239	8204	AAGAGCAGAGATTGGATGGAAGGG	52.0	58.0	25	-3.1	23.5	0.05
MHC_66_30246418	30246418	30246443	4853	CTCCTGTCTTCCACCCATCTCCATC	56.0	58.1	25	-2.0	12.8	0.74
MHC_67_30251271	30251271	30251296	11655	GTCGGCTTACTTTGCTCTGGATGTG	52.0	58.2	25	-3.6	27.5	-0.21
MHC_68r_30262926	30262926	30262951	8049	TGTGGCAGAGACATGAAACAGATGC	48.0	57.8	25	-5.4	25	-0.01
MHC_69_30270976	30270976	30271000	6788	CCACACAGTAGGCATTCACACATGG	52	58	25	-5.3	22.7	0.18
MHC_70b_30277764	30277764	30277788	9213	AAGCCCTTTATCATGCCTCTTTGCC						
MHC_71_30286976	30286976	30287001	7981	ACATTCTTGCCCACACACATTACCT	44.0	56.9	25	-3.1	18.3	0.43
MHC_72_30294957	30294957	30294982	7354	ATGGAAGATTCAGGTATTGTCAGCC	44.0	54.9	25	-3.5	28.9	-0.32
MHC_73r_30302311	30302311	30302336	8574	CTG TCC CAT GCT GCC TTC CCT AAA G	56.0	59.3	25	-5.4	28.1	-0.43
MHC_74r_30310885	30310885	30310910	7775	CTGCTGCTTTATGGAGACAAGGTGG	52.0	57.9	25	-3.5	30.4	-0.27
MHC_75r_30318660	30318660	30318685	1590	CCTATGCCCACCTGCCAACACATAT					22.3	0.16
MHC_76_30320251	30320251	30320275	14292	GCAAACACTTCTATGGCTCACAGGG	52	58	25	-3.1	18.3	0.36
MHC_77_30334542	30334542	30334567	8571	AGGGAAACTCGGGCTATGGAATGAA	48.0	57.7	25	-3.6	22.9	0.07
MHC_78_30343113	30343113	30343138	5243	ATTCCAGCATCTACCTCGGTGTCAG	52.0	58.1	25	-4.4	29.2	-0.29
MHC_79_30348356	30348356	30348381	16134	TTTGGGCTTACAACAGGATGGCAAC	48.0	58.2	25	-3.9	30.2	-0.31
MHC_80_30364490	30364490	30364515	1273	ACCCAACCCACAGTAGATACCAACA	48.0	57.3	25	-2.3	12.7	0.42
MHC_81_30365764	30365764	30365788	6495	GTGCTCAATCCCACCACACATCTTG	52	58	25	-3.9	28.5	-0.27
MHC_82_30372258	30372258	30372283	10741	TCAGAGGTCAGAAGGGTGTTTGCTG	52.0	58.6	25	-3.6	22.5	0.19
MHC_83b_30383000	30383000	30383024	7313	ATTCTACAGGTCCCTTGCTCCCTTG						
MHC_84r_30390312	30390312	30390337	9420	ATGCCTGCTGTCTGGTACTATGCTT	48.0	58.1	25	-3.6	21.5	0.26
MHC_85r_30399733	30399733	30399757	6648	GGATGAAGGCGGGGTTTAATTGTCC	52	58	25	-5.3	12.3	0.75
MHC_86r_30406380	30406380	30406405	8315	CCTTCCTTGCCTGTCATTTGGGATG	52.0	58.1	25	-4.6	26.2	-0.1
MHC_87r_30414695	30414695	30414720	8143	GCCTCACTGCTTCAAATGCTGGTAA	48.0	58.0	25	-3.1	25	-0.03
MHC_88_30422838	30422838	30422863	8118	TGAAGAGCTGGAGAGAATGATGCCA	48.0	57.8	25	-6.3	23.7	0.12
MHC_89_30430956	30430956	30430981	8514	TTGTATCCCAAACGGTCCCATCTCT	48.0	57.5	25	-3.9	25	0
MHC_90_30439471	30439471	30439495	6117	GCTGGTGGTGCTCAATGGTTGTTAT	48	58	25	-3.9	27.7	-0.17
MHC_91_30445588	30445588	30445612	8641	CTAGCTGGATCAGTGGGCTTCTAGG	56	58	25	-6.3	34.8	-0.92
MHC_92r_30454228	30454228	30454253	11416	GTAGGGAGCCAGATCCTCTTTCACC	56.0	58.1	25	-4.7	32	-0.63
MHC_93_30465646	30465646	30465669	4659	GACTCTATAACCATACCCAGGGCA	50	58	24	-6.1	26.4	-0.1
MHC_94_30470303	30470303	30470328	8193	AGCCTTATTGCAGGAGAGGAAGGTG	52.0	58.3	25	-7.0	34.5	-1.11
MHC_95_30478496	30478496	30478521	11857	AATGCCCCTCAGAATAACCCTCCTC	52.0	57.8	25	-3.1	12.9	0.68
MHC_96_30490353	30490353	30490378	4434	ACACTTAGCCACTTCTGCTTCCTCA	48.0	57.9	25	-4.7	33.7	-0.7
MHC_97_30494787	30494787	30494812	5556	GTGGAGGCTGGAGAAACTAGGATGG	56.0	58.4	25	-4.2	21.6	0.29
MHC_98_30500343	30500343	30500368	14004	TATCTTAGTGCCCAGTTGAAACCTC	44.0	54.6	25	-3.3	20.5	0.25
MHC_99r_30514347	30514347	30514372	4801	TCCAGTGAGTGAGAGGAGGGATGAA					29.4	-0.25

MHC_100_30519148	30519148	30519173	7458	TCGGTTAATGTCTGTACCCTCCACT	48.0	56.9	25	-4.9	23.8	0.07
MHC_101_30526606	30526606	30526631	6858	GACCAAGTAGGCTCTAACCACGGAA	52.0	58.0	25	-3.6	37.6	-0.71
MHC_102_30533462	30533462	30533489	9618	TACCTGTCTCTGTATAGGCAAAACTGT	40.7	55.6	27	-4.7	29.9	-0.45
MHC_103_30543082	30543082	30543107	3904	CTGGAATAACTGAGGCTGAACGCAC	52.0	58.2	25	-3.6	40.2	-1.1
MHC_104_30546986	30546986	30547011	10070	GCCTTGTATTTCCCAGCACAATTTC	44.0	55.5	25	-5.4	24.1	0.05
MHC_105_30557060	30557060	30557081	9684	CGCAGAGGAAGACAGACCCCTA	59	60	22	-4.6	29.7	-0.31
MHC_106r_30566740	30566740	30566765	6623	AGCCTGGACTTAGGGATCTGGGATA	52.0	57.8	25	-4.7	29.4	-0.42
MHC_107_30573364	30573364	30573388	10065	AATCACCTATGCTTCCTCAGTGGTA	44	57.4		-4.4	32	-0.54
MHC_108r_30583428	30583428	30583453	7611	TGG AAT CTG GTG CCT GTG GTC TTA T	48.0	57.7	25	-3.1	22.2	0.19
MHC_109_30591039	30591039	30591064	5362	CGTAGTTCTTCACAGAGGGCTCCAT	52.0	58.1	25	-3.6	23.5	0.06
MHC_110_30596401	30596401	30596426	7447	GGATGCTCGCCAATCTCCTCTGAAT	52.0	58.7	25	-4.6	20.5	0.37
MHC_111_30603848	30603848	30603873	13366	AATGAGAAACCATTGTCCCAAGACTG	42	57.4	26	-5.3	38.9	-1.27
MHC_112_30617214	30617214	30617239	5437	GCTACTGACACAAGAACGACAATGC	48.0	57.0	25	-3.6	21.7	0.2
MHC_113_30622651	30622651	30622676	8068	CCACTCCCATAACACAGTCCTTCCA	52.0	57.9	25	-2.9	0.5	1.25
MHC_114_30630719	30630719	30630744	8163	AAGAGTACATCCCAGACCCCTCCTT	52.0	58.1	25	-3.6	16.6	0.48
MHC_115_30638882	30638882	30638907	7739	TCCCCTTGTAGCCTTTCTCAGTGTC	52.0	57.9	25	-3.1	15.9	0.51
MHC_116_30646621	30646621	30646646	8244	ATTGGTCTGCACTGTCTTTTCTGCC	48.0	58.0	25	-7.0	30	-0.51
MHC_117_30654865	30654865	30654890	7377	CTACTTGTGACTCTTCTGCGTGTGC	52.0	58.1	25	-3.6	33.7	-0.52
MHC_118_30662242	30662242	30662267	4483	GCTACCAGGGACAGAAACCACTCTT	52.0	58.2	25	-3.2	27.5	-0.19
MHC_119b_30666726	30666726	30666750	12113	ATTGTGAGGTTCCATCCAGCCAAGT						
MHC_120_30678838	30678838	30678863	7490	GTCTTCCCCTCCCATTTCTTGCCTA	52.0	58.0	25	-3.1	12.1	0.73
MHC_121_30686328	30686328	30686353	8123	GGAGTAGGTGCCAGAGATTGAGACA	52.0	57.5	25	-3.1	26.7	-0.12
MHC_122r_30694451	30694451	30694476	7775	ACAGGGCAGAACAAACTTGAGTCCT					30.3	-0.37
MHC_123_30702226	30702226	30702251	8541	GATGCTGTGGGGAGATAAGTGGTCA	52.0	58.1	25	-3.1	19.2	0.36
MHC_124_30710767	30710767	30710792	8236	GTCTCGTTCTGAGCATTCCAGTAGC	52.0	57.5	25	-3.6	37.1	-0.95
MHC_125_30719003	30719003	30719028	7941	TGTGGAATGGTAGGAAGAACACCGA	48.0	57.6	25	-4.4	27.1	-0.21
MHC_126_30726944	30726944	30726969	8030	TGTTCCCCATTCCTTAGAAGCCAGT	48.0	57.6	25	-3.5	25	0.02
MHC_127_30734974	30734974	30734999	7599	TCCCACCTCTTAGCACATCAAGGAC	52.0	58.0	25	-4.7	36.6	-1
MHC_128_30742575	30742575	30742598	8381	GCCCCTCTGCTGTTCTACCCTTA	56.5	57.6	23	-3.1	19.9	0.34
MHC_129_30750954	30750954	30750979	8040	TGACACCTGGCTTGTTCTCTTGCTA	48.0	58.1	25	-3.3	27.5	-0.21
MHC_130_30758994	30758994	30759019	7510	AGATGTGTGTGACTGGAGGGACTTC	52.0	57.9	25	-1.6	32	-0.18
MHC_131_30766504	30766504	30766529	9169	TTCCCCTGTCATTTGTTTCCAAGCC	48.0	57.9	25	-3.9	18.1	0.49
MHC_132_30775673	30775673	30775698	7216	CTAAAGATGCTTCCAGAGGCCAGGA	52.0	58.1	25	-9.3	25	-0.04
MHC_133r_30782889	30782889	30782914	7726	GTTTCTTCTTTCTTTGCCCCAGCCA	48.0	58.1	25	-3.1	31.5	-0.49
MHC_134_30790615	30790615	30790640	7971	TCCAGTGAGTCCAGGGTAGTAGTCT	52.0	57.4	25	-3.1	20.3	0.35
MHC_135_30798586	30798586	30798611	8239	CTCTGATCCCTGCTGTCTCCCATTT	52.0	58.0	25	-4.6	4.2	1.04
MHC_136_30806825	30806825	30806850	7992	TGCTCCTCAATCTGCTGCTTAGTCT	48.0	57.8	25	-3.1	28.6	-0.29
MHC_137r_30814818	30814818	30814842	7726	GCAGCCCTTGATATTGCCAGGAAG	54.2	58.1	24	-5.1	31.8	-0.55
MHC_138_30822543	30822543	30822568	9115	TCAGTATAAGTGCTGTGCCATGCAG	48.0	57.4	25	-7.0	37.3	-1.12
MHC_139r_30831658	30831658	30831683	7795	GCTCTATTCCAGCCCATCTCTGTCC	56.0	58.5	25	-4.7	25	-0.03
MHC_140r_30839453	30839453	30839478	6947	AGG AAA GAA AGG AGC AGA TGT GGC A	48.0	58.4	25	-3.1	25	0.01
MHC_141_30846400	30846400	30846425	8154	AGTGTTAGGTAAAGTGTGGAGGAGA	44.0	54.8	25	-2.9	-21.8	1.56
MHC_142r_30854554	30854554	30854579	9115	CAA AGA AAT GCT GCC CCA ACA CAC T	48.0	58.6	25	-3.1	10.2	0.93
MHC_143r_30863669	30863669	30863694	6908	TTG GGA TCA TAC AGC AGA GAC AGC A	48.0	57.8	25	-4.6	28.6	-0.29
MHC_144_30870577	30870577	30870602	8088	GACAACTAATAAAGCCCTGTGCCCC	52.0	57.9	25	-3.3	35	-0.57
MHC_145_30878665	30878665	30878690	7587	GCCTTCTACAGACAAGCCAATGTGA	48.0	57.3	25	-3.5	30.9	-0.44
MHC_146_30886252	30886252	30886277	8326	GAGAATCGGTTACTAGGCCGTATGA	48.0	55.8	25	-9.3	43	-1.93
MHC_147_30894578	30894578	30894603	8290	ACTGTCCTGTACTTAGCCTTGCTCT	48.0	57.2	25	-4.7	34.7	-0.7
MHC_148_30902868	30902868	30902893	8103	CTAGGTCTTATATGGCAGTCGGGGC	56.0	58.3	25	-4.2	18	0.42
MHC_149_30910971	30910971	30910996	8660	GGTGTGTCTTGAGCAGAGGTTGAGA	52.0	58.4	25	-3.2	29.9	-0.34
MHC_150r_30919631	30919631	30919656	6279	CCACCTTTGGCTTTTCTAGGCTTTG	48.0	56.8	25	-5.0	30.6	-0.43

MHC_151_30925910	30925910	30925935	8161	AGACAGGTAGCATGAGTTCTTGGCA	48.0	57.9	25	-5.4	29	-0.34
MHC_152_30934071	30934071	30934096	7747	CTGGTGAGAATGGAGGTGTGTAGTG	52.0	56.8	25	-1.6	3.2	0.92
MHC_153_30941818	30941818	30941843	9075	CACAAAACAGCCACCCTCTACTTCC	52.0	57.7	25	-3.1	-13.5	1.21
MHC_154_30950893	30950893	30950918	7535	TGACTGCCCTCTGACTTCTTCACTT	48.0	57.6	25	-3.5	23.5	0.14
MHC_155_30958428	30958428	30958453	7934	AGAGGGGACAAAGGTTTCAGGACTC	52.0	57.9	25	-3.9	6	0.66
MHC_156r_30966362	30966362	30966387	8191	CCT TCA TTT CAG CCC ATT CCC TCT G	52.0	57.6	25	-3.6	27.8	-0.16
MHC_157_30974553	30974553	30974578	7879	CAGAGTGGATCTGGGGCTTCCAATA	52.0	57.8	25	-6.6	43.3	-1.75
MHC_158_30982432	30982432	30982457	7853	GGCACTTCCTGTCTGATTGTAGGGA	52.0	58.0	25	-4.7	28.5	-0.35
MHC_159_30990285	30990285	30990310	7994	ATCATCTCCCTGAGTTTCTGAGCCC	52.0	57.8	25	-3.5	26.3	-0.11
MHC_160_30998279	30998279	30998304	8207	CAAGAGACATCATCAGTGGGGTGGA	52.0	58.0	25	-2.0	26.5	-0.12
MHC_161_31006486	31006486	31006511	8376	TCACAAGCTGAACCCGTCATCTCTC	52.0	58.5	25	-6.3	26.4	-0.1
MCH_162b_31014862	31014862	31014887	8112	CCAGGTTTCTCCATCCCAGACATCA	52.0	58.0	25	-3.2	39.5	-1.07
MHC_163_31022974	31022974	31022999	7509	CAGGTAATGGGATTGGGACAGTGGA	52.0	58.0	25	-3.4	27.3	-0.1
MHC_164_31030483	31030483	31030508	8339	TCAGTCCCAACAGAAACCTCCCATC	52.0	58.2	25	-1.6	25	0.01
MHC_165_31038822	31038822	31038847	8942	CAACTTTCACCACCTGAAGAGGCTG	52.0	58.0	25	-5.5	39.4	-1.09
MHC_166_31047764	31047764	31047789	6819	CCTCTATTCTGGTGACTGGTGCTGT	52.0	58.0	25	-3.1	3.2	0.94
MHC_167r_31054583	31054583	31054608	7649	AGA TGA AGA ACT GGA GGG CAC TGA G	52.0	58.0	25	-3.1	4.2	1.04
MHC_168_31062232	31062232	31062257	7558	GGGATCAGCATAGCCACCAACTCTG	56.0	59.2	25	-4.6	23.6	0.1
MHC_169_31069790	31069790	31069815	8844	CCTACAACTTCCTGCCCCTACATGG	56.0	58.6	25	-5.4	28.4	-0.16
MHC_170_31078634	31078634	31078659	7867	GGAACTGTGGAAACCCTTATGTGGA	48.0	56.7	25	-3.1	34.8	-0.61
MHC_171_31086501	31086501	31086526	9161	GTGGCTGCTTCTCTTTGGACTTCTG	52.0	58.0	25	-3.1	4.2	0.92
MHC_172_31095662	31095662	31095687	6825	CTTGTGAGGGTGAGAGGGAGTATGG	56.0	57.8	25	-1.6	6.8	1.03
MHC_173_31102487	31102487	31102512	8586	TCGGAAACCATCACACCCTCTACTG	52.0	58.0	25	-3.6	29.3	-0.25
MHC_174_31111073	31111073	31111098	7536	TCCCTGTGTTTCCTCATGGTGCTAT	48.0	57.7	25	-5.4	29.2	-0.35
MHC_175r_31118609	31118609	31118634	6293	TGTTCCTTCACAGAATGCCACCTCT	48.0	58.1	25	-3.5	23.9	0.12
MHC_176r_31124901	31124901	31124927	9546	TCG TCC TCC CTT AGT GTG ACT TAT CC	50.0	57.9	26	-3.6	18.7	0.46
MHC_177_31134448	31134448	31134473	7863	CTCAGGGTGTGGGAAGTAAGAGGAA	52.0	57.4	25	-3.2	18.4	0.39
MHC_178r_31142311	31142311	31142336	7895	TGCCACCTCCTTTCATGGGTAGAAG	52.0	58.2	25	-5.4	27.9	-0.22
MHC_179_31150206	31150206	31150231	8164	CATTCCTCACCTCTGTCTTCTACCA	48.0	55.5	25	-2.0	18.7	0.36
MHC_180_31158370	31158370	31158395	7878	AGGAAGAGAGAATTAGGGGTTGGTA	44.0	54.4	25	-5.4	-2.3	1.25
MHC_181r_31166248	31166248	31166273	7484	GTC ACC CAG CAA CGT ATT TTG ATT G	44.0	55.5	25	-6.3	21.1	0.34
MHC_182r_31173732	31173732	31173757	9269	CCAAGTCAACCCAATTCTTCATCTT	40.0	53.8	25	-5.4	12.4	1.11
MHC_183_31183001	31183001	31183026	7785	TGTAACTCCCAGTGCAAAGCATCAC	48.0	57.8	25	-7.0	29.4	-0.3
MHC_184_31190786	31190786	31190811	7972	CTGGAGCAATGAGAGAGGAGGGAAA	52.0	57.8	25	-3.1	-13.7	1.69
MHC_185_31198758	31198758	31198783	8236	TTCCAACACAAACTTCCCCTGACCA	48.0	58.4	25	-2.0	15.3	0.62
MHC_186_31206995	31206995	31207019	7665	CATTAAGTGTGCATGACGGCCCAG	54.2	58.6	24	-9.3	28	-0.17
MHC_187_31214659	31214659	31214684	8092	TTCACCTCCGCCATCTTGAGTATCC	52.0	58.1	25	-3.6	23.9	0.07
MHC_188_31222750	31222750	31222776	8189	GACTCATCCTGCATCTTAATTTCGCT	42.3	55.7	26	-7.0	25	0.03
MHC_189_31230940	31230940	31230965	8095	GGAAAGGTTGGAAGAACACACAGGG	52.0	57.9	25	-3.3	36.2	-0.97
MHC_190b_31239035	31239035	31239060	7516	CTTGCTTTCCAGGGAGAATGTGCTT	48.0	57.7	25	-4.6	22.7	0.19
MHC_191_31246551	31246551	31246576	8581	CTAATGGTGGTGGCAATGGTGTCTG	52.0	58.1	25	-3.1	20.7	0.33
MHC_192_31255132	31255132	31255157	8001	CCACGCTGCTGTACCCCTTTATTTC	52.0	58.2	25	-3.6	21.3	0.22
MHC_193_31263133	31263133	31263158	7544	AGGGTTTGTGGTTTAGGAAGTGAGC	48.0	57.1	25	-3.1	-29.5	1.7
MHC_194_31270677	31270677	31270702	7718	CAAACCCCACAATGACTTCTGCCAT	48.0	58.0	25	-3.4	9.2	0.67
MHC_195_31278395	31278395	31278420	11764	AGTGTTTTCAGAGGACCAGGGTTGA	48.0	57.7	25	-4.4	23.3	0.07
MHC_196_31290159	31290159	31290184	1648	AAGGTTGTTGTGAAGGTCATCTGTG	44.0	55.7	25	-3.5	11.9	0.49
MHC_197_31291806	31291806	31291832	9179	ATAGACTTGAACTGACCCAATGCCCA	46.2	58.0	26	-3.9	15	0.7
MHC_198_31300986	31300986	31301011	11055	TAGTGGATTGTGGATTGAGGTGCGA	48.0	58.0	25	-3.6	-9.9	1.31
MHC_199_31312039	31312039	31312066	6322	ACCCCTGTTTATCTCTGTTATCTCTGG	44.4	55.7	27	-3.1	-1.9	1.25
MHC_200_31318363	31318363	31318388	8517	ACTCCGCATCTGCTTCCTAAGACAT	48.0	57.8	25	-5.1	36.1	-0.89
MHC_1_31326881	31326881	31326905	13174	GGACAAAGGCTAATTTCCTCAGAAT	40.0	53.4	25	-5.4	21.5	0.19

MHC_2_31340054	31340054	31340079	2187	GTCCCTAAGTCTGCTAAACACAGGTT	46	58	26	-4.6	30.6	-0.49
MHC_3_31342241	31342241	31342266	7741	TATACATCCTCCCTTTCCTTTCTGC	44.0	54.1	25	-3.4	-2.4	1.76
MHC_4_31349982	31349982	31350007	12302	TGCCCAGTAGAGAAACACCAAATGC	48.0	57.7	25	-3.1	23.3	0.06
MHC_5r_31362284	31362284	31362309	4248	TGT TCT AAA GGA GGC CAC AGG AGT TC	50	60	26	-9.2	23.3	0.07
MHC_6r_31366532	31366532	31366557	8070	CAT AGA ACC TTG GGG TCA CAG ATG C	52.0	57.4	25	-4.4	36	-0.76
MHC_7_31374602	31374602	31374627	8510	GGGGAGATGAGGTGATACTGGGTTT	52.0	57.5	25	-1.6	6.8	0.81
MHC_8_31383112	31383112	31383137	7768	AACCTAGCAGTCCTTCGTGGTACTT	48.0	57.4	25	-4.4	32	-0.54
MHC_9_31390880	31390880	31390905	4494	GGGTGAGACTTTCTTGAACTCAGCA	48.0	56.9	25	-5.1	33.4	-0.86
MHC_10_31395376	31395376	31395399	8634	CCCAGCCAACACATAGAACCCTA	52.2	55.9	23	-3.1	19.9	0.35
MHC_11_31401206	31404012	31404033	14009	ATGGTTCATGGATGTTAGGGC						
MHC_12_31412706	31418015	31418042	12767	ACCCAGAATTATAGAGTGAACGTGAGA						
MHC_13r_31430785	31430785	31430809	7765	TCCCTTGTGAAGATGGACCCCGAA	54.2	59.0	24	-4.7	27.7	-0.2
MHC_14_31438549	31438549	31438574	11925	GTCTTGCTCTTCCACTCTACCCAGT	52.0	57.7	25	-3.1	12.8	0.78
MHC_15r_31450473	31450473	31450499	3878	GTT AGC CCC AGA GTT GCA TCT GAG CA	53.8	62	26	-7	36.4	-1.34
MHC_16_31454352	31454352	31454377	5454	CGTCAGTTATGGAATGTGGACCCCT	52.0	58.3	25	-3.6	21.6	0.27
MHC_17_31463174	31459808	31459831	11024	GCTGAGAATGACAGAGCACGGGAG	58	61.4	24	-4.7	37.6	-1.18
MHC_18_31470830	31470830	31470855	7957	TCGCTTTGTGTAGGAAGGATCAGGA	48.0	57.5	25	-4.6	26.4	-0.06
MHC_19_31478787	31478787	31478812	8415	AGTTTGTGGTCTGAGCGGTTCTACT	48.0	57.9	25	-3.6	22.2	0.19
MHC_20_31487202	31487202	31487227	5327	TGTGCTATGGATGAAGGCATTTCCTG	46	59	26	-5	43	-1.15
MHC_21r_31492531	31492531	31492554	5258	TCT CTG CCA TGA CCA CCT TAC GTG						
MHC_22_31497789	31497789	31497812	15769	ATGGTGTCAGAAAGAGCCCTGAAG						
MHC_23r_31513556	31513556	31513581	5133	TCACAACAACTTCTCACTACCGATT	40.0	54.7	25	-3.1	10.6	0.61
MHC_24_31518689	31518689	31518714	6763	CGCACTTCCCTCACTTTGATACCCT	52.0	58.5	25	-3.6	10.9	0.63
MHC_25_31525452	31525452	31525477	8974	GGACCGTGACCTTGGAGTAAGAACC	56.0	58.8	25	-3.6	36.4	-0.85
MHC_26_31534426	31534426	31534451	9648	AGGGAGAGTTTGTTGATGATTGGTT	40.0	54.6	25	-1.5	-81.9	1.86
MHC_27_31544054	31544075	31544099	6714	GGCACCCATCTGATAATGCTGTCCT	60	52	25	-5	35	-0.99
MHC_28_31550788	31550788	31550813	6615	GTATCATCCCATCCACCAGTGCCTT	52.0	58.3	25	-3.3	21.9	0.21
MHC_29b_31557403	31557403	31557428	9295	AGTGAGGACCAATGAGTTGACAGGT	48	57.6	25	-4.4	35.5	-0.9
MHC_30_31566698	31566698	31566723	6651	TCCCTGAGTGTCCTTGACTTTCCTC	52.0	57.6	25	-3.2	32.5	-0.55
MHC_31r_31573350	31573350	31573374	8959	TCA GAA GCA CTT GCC CCA GTA GAA A	48	60	25	-5	36.2	-0.8
MHC_32_31582308	31582308	31582333	8106	TGTGTCCTGATTTGCCTCTCCTGTT	48.0	58.1	25	-3.1	10.2	1.06
MHC_33_31590414	31590414	31590439	8337	ACACCTGACCTACATTTCTTCAGTT	40.0	54.1	25	-5.1	32.1	-0.78
MHC_34_31598751	31598751	31598776	8382	TGGCTAAACTTCAACATCCCTCAGC	48.0	57.3	25	-4.7	21.3	0.29
MHC_35b_31607133	31607133	31607158	7376	CGCTGCACAGACTTCACAAAGATCA	48.0	58.1	25	-7.0	39	-1.1
MHC_36_31614509	31614509	31614534	8199	CTTGGCTTACCTTGACATTGGGCAT	48.0	57.8	25	-3.1	33.8	-0.61
MHC_37_31622708	31622708	31622733	8218	GGGTTGTGAATGCGGTGAAAAGGTA	48.0	58.0	25	-3.6	-7.9	1.78
MHC_38_31630929	31630929	31630951	8225	CCTACAGCCCCACCACCCATAG	63.6	58.1	22	-3.1	4.4	1.64
MHC_39_31639151	31639151	31639176	4977	TGAAGAACATCAGAGGCACGGAGAT	48.0	57.8	25	-3.6	21	0.35
MHC_40b_31644129	31644129	31644153	10317	CTAGAAACAGGGTTGGGTTGGGGTA	52	60	25	-4	25	-0.03
MHC_41_31654446	31654446	31654470	8197	CACAGCCAAGGGACTTCCAGAGAT	54.2	58.1	24	-4.6	38.1	-0.96
MHC_42_31662642	31662642	31662667	6217	CCTTACCAGCACCTAGAACCATCCA	52.0	57.8	25	-4.2	15.2	0.55
MHC_43r_31668859	31668859	31668884	6341	TCCTCTGGTCGAAAATGGGTCTTGT	48.0	57.6	25	-6.3	21.6	0.22
MHC_44b_31675201	31675201	31675225	11876	TAAACTTCCAGACCCCATTGCCCTA	48	59.8	25	-3.14	20.9	0.18
MHC_45r_31687076	31687076	31687101	7702	CGGGTCAGTCGTAGAGAACAAGTAG	52.0	57.9	25	-3.1	21.7	0.22
MHC_46_31694778	31694778	31694803	7639	TTCTCAATCACCCCAGGCATAATCA	44.0	56.0	25	-3.1	13.2	0.55
MHC_47r_31702417	31702417	31702442	7805	GCCTCCCTTCCTATAACCGTAACCA	52.0	58.6	25	-3.9	5	0.83
MHC_48_31710222	31710222	31710247	8103	CTCCAGCCACTAGCCGAAAGAGTTA	52.0	58.1	25	-4.2	36.7	-0.66
MHC_49_31718325	31718325	31718350	8450	CAGGCAGCACAACTTACCTACCTCT	52.0	58.2	25	-4.7	26.8	-0.1
MHC_50_31726775	31726775	31726800	8204	AGACACACTTGCTTAGGGTTCCTGT	48	57.8	25	-4.7	33.3	-0.63
MHC_51_31734979	31734979	31735004	7458	TGGGCAGTAAGTAGGCTCAGAACAG	52	58	25	-3.1	26.4	-0.12
MHC_52_31742437	31742437	31742462	8399	GAGTGCTTCAGAGTTTGGGTTGCTT	48	57.9	25	-3.1	23.8	0.1

MHC_53_31750836	31750836	31750861	8240	AGATGAGTATAGCCAGGTGTCTGCC	52	57.7	25	-3.6	33.7	-0.71
MHC_54_31759076	31759076	31759101	7261	TGTCCCTTCTGAGTGCTCCTACCTA	52	57.9	25	-3.2	23.6	0.09
MHC_55r_31766337	31766337	31766362	8278	TAG GTG ATC TGC TGT GAG GGG AAT G	52	57.8	25	-4.6	30.4	-0.32
MHC_56_31774616	31774616	31774640	7729	TGTTGAAGCATCGCCCATCACCTG	54.2	59.8	24	-3.6	17.8	0.39
MHC_57_31782344	31782344	31782369	7959	CAG AGA AGT GGT CCT GCC TGT CTA G	56	58.1	25	-4.2	35.9	-0.77
MHC_58_31790303	31790303	31790328	7980	TTC CTC CTT CCC AGT CTC TTC AAC C	52	57.6	25	-1.9	9.3	0.97
MHC_59_31798284	31798284	31798308	8563	CTT CTA CCT CAC CTC TCT GTG CCC	58.3	58	24	-3.3	28.9	-0.3
MHC_60_31806846	31806846	31806871	7743	AACCAGCTCAACTCCTCACTGTCTT	48	57.8	25	-6.3	27.2	-0.18
MHC_61_31814589	31814589	31814614	8524	TCCATTGCTCAACAGATTTGCCAGG	48	58	25	-3.9	26.4	-0.1
MHC_62_31823113	31823113	31823138	5809	CAAGTTCACTCCCTGCCTTATCCCT	52	58	25	-3.5	5.8	0.78
MHC_63r_31828922	31828922	31828947	9733	CGA TGT GCT ATG GGA ATG TAG TGG C	52	57.8	25	-3.6	15.4	0.61
MHC_64_31838655	31838655	31838680	8365	TCTTCTCAAGTAGGAACACCCCAGC	52	57.9	25	-3.5	28.3	-0.18
MHC_65_31847020	31847020	31847045	7207	AAT GCC TAC TGG ACT GAG AGT TGC C	52	58.5	25	-3.1	40.3	-0.99
MHC_66_31854227	31854227	31854252	7174	GTACAGAGTCCAGCTTCCAGACCAG	56	58.3	25	-6.3	26.2	-0.12
MHC_67_31861401	31861401	31861426	9113	ATAGCTTCTTCTCCACCACCACCTG	52	58	25	-6.3	2.4	1.06
MHC_68_31870514	31870514	31870539	6608	TCATTGTCCATTCCAGTCCTCGCTT	48	58.2	25	-3.6	-13.9	1.47
MHC_69_31877122	31877122	31877147	9535	AAACATTTGACAGCACCCCACCATC	48	58	25	-3.8	5.4	1
MHC_70_31886657	31886657	31886682	7068	CGG CTG CTT CAG GAC TAC TTC AAT G	52	58	25	-3.6	29.8	-0.37
MHC_71b_31893726	31893726	31893750	9338	GGTCAGTAGGGTCTGCATAGGTTGT	52	57.8	25	-7	29.6	-0.45
MHC_72_31903063	31903063	31903088	8115	CCAATCAGAGTTTCCCAGGCACATC	52	57.9	25	-3.1	10.4	1.02
MHC_73_31911178	31911178	31911203	7887	CGTTCATCGCCGTGTTATCCTTGAG	52	58.3	25	-3.6	28.6	-0.16
MHC_74_31919065	31919065	31919090	8087	AGATAACAGAGTTCCTACCCACCCT	48	56.1	25	-3.3	19.4	0.38
MHC_75_31927152	31927152	31927177	7729	TGGGAGTGGGGATCAATGTGTGAAG	52	58.4	25	-4.6	29.5	-0.33
MHC_76_31934881	31934881	31934906	9195	GAACTCACCCAGGCTCACAAGGATA	52	58	25	-3.1	28.2	-0.18
MHC_77_31944076	31944076	31944101	6646	GGCTCTGTGTTCCAAGGGAGTAAGA	52	58	25	-4.6	27.9	-0.27
MHC_78_31950722	31950722	31950747	7725	GAGATGATGTTGCTGGACAGGATGC	52	58	25	-3.6	27.6	-0.17
MHC_79_31958447	31958447	31958472	9224	GGG ACC TTC ATC TGC GAG TAA GTC A	52	58.1	25	-3.6	28.1	-0.25
MHC_80_31967671	31967671	31967696	7133	CCTTTGTGGATGAAACGGGCAGAAT	48	58	25	-3.9	33	-0.45
MHC_81_31974804	31974804	31974829	7576	CGCAGTTGCCAGAGTGTAAAGGAAA	48	58	25	-5.1	33.2	-0.58
MHC_82_31982380	31982380	31982405	8719	CCAAGTCTGCTCTGCTACCCTATGT	52	57.8	25	-3.1	25	0.01
MHC_83_31991099	31991099	31991124	7724	GGTTTCATTGCTCACCTGGATGCTT	48	58	25	-4.6	28.6	-0.29
MHC_84_31998823	31998823	31998848	6003	AAACACGGTTACATTTGCTGGGCTT	44	58.1	25	-3.9	25	-0.03
MHC_85b_32004826	32004826	32004851	10064	CAGGTCACACATCACAAGTCTGCAA	48	57.7	25	-7	14.2	0.59
MHC_86_32014891	32014891	32014915	7825	ATGTTGAGGTTCCCAGGCTAAATGC	48	57.5	25	-3.1	34.7	-0.63
MHC_87_32022716	32022716	32022740	8042	GCAGCCTTTCCCTCTTGATGACTTC	52	57.9	25	-3.1	5.4	0.75
MHC_88_32030757	32030757	32030782	8066	CAAGGACTCAGGCAATCAACTCCAC	52	57.8	25	-4.6	27.3	-0.17
MHC_89_32038823	32038823	32038848	8227	CCCTTGAGTTTGCTGACTGGATTGG	52	58	25	-3.1	28	-0.17
MHC_90_32047050	32047050	32047075	7556	TATACTGCCTACCACGCTTTGCTCA	48	58.1	25	-3.6	22.3	0.21
MHC_91_32054606	32054606	32054631	5574	CAGAGCATCTTACCCTGATACGCCT	52	58	25	-3.6	25	-0.04
MHC_92_32060180	32060180	32060205	11000	TTATTGTAACCCCAGACCCTTGCCT	48	57.6	25	-3.1	2.6	1.3
MHC_93_32071180	32071180	32071205	7383	AACCATGATCTACTTGGCTCCGACA	48	57.8	25	-5.4	32.2	-0.55
MHC_94r_32078563	32078563	32078588	7700	TAG CGG GTC ATC TGG GAG GTT CTA G	56	58.9	25	-4.2	9.2	0.67
MHC_95_32086263	32086263	32086288	6296	GTAGTTGGTGTGGAGGACAAGGTCA	52	58	25	-3.9	31.9	-0.64
MHC_96_32092559	32092559	32092584	9900	ACGACAGGTACATCTATGGGAAGCC	52	58	25	-3.6	30.1	-0.38
MHC_97r_32102459	32102459	32102484	8056	TCA CCT TTG GTT TTG GAC AGG CTC A	48	58.7	25	-4.7	29.3	-0.34
MHC_98_32110515	32110515	32110540	8482	GGCGAAGGTGGAATGAGAGGATCTA	52	57.7	25	-4.6	38	-0.61
MHC_99_32118997	32118997	32119022	8064	TGTAGTTGGTGTGGAGGACAAGGTC	52	58.1	25	-3.9	26.4	-0.11
MHC_100_32127061	32127061	32127086	7563	CGTTAGCCCCATTAGGACAGGAACT	52	58.1	25	-3.6	39.5	-0.77
MHC_101_32134624	32134624	32134649	8544	ATC ATT CAC CTC ACG GCT ACA CAA C	48	57.4	25	-3.6	28.4	-0.15
MHC_102b_32143169	32143169	32143193	5819	AGAGCCACAAACCAGCCAGTGAAT	50	58.8	24	-3.3	32.1	-0.67
MHC_103_32148987	32148987	32149012	9445	GGACTCAGGATGTAAAGCACTTCGC	52	58	25	-3.6	32.9	-0.42

MHC_104_32158432	32158432	32158457	8224	CTTCCCAGTACAACCTCCACTGCTT	52	58.4	25	-4.9	33.7	-0.9
MHC_105_32166656	32166656	32166681	8249	CAATACACACACCTTGGATGCTCCC	52	57.9	25	-4.6	33.6	-0.61
MHC_106_32174907	32174907	32174930	7596	GCCAATCCTTCCCCAAACACCTG	56.5	58.1	23	-3.1	-6	1.63
MHC_107_32182501	32182501	32182526	8299	CCTGGAGTGTAGCCTTGGGTAAGAG	56	58.1	25	-3.5	33.8	-0.66
MHC_108_32190800	32190800	32190825	6118	CGTATCCTCTAGTCTGTCACGCCAT	52	57.8	25	-5	19.6	0.42
MHC_109_32196918	32196918	32196943	8867	GTGTCGAGGAGAAGGAGCTGAAGAA	52	58	25	-6.7	7.5	0.66
MHC_110_32205785	32205785	32205810	9730	TCCACAATTCCCTGCGGTTAAGGTA	48	58	25	-5.3	28.4	-0.32
MHC_111_32215516	32215516	32215540	7243	GATGTCCCACAGGAGATACTTGGC	54.2	56.8	24	-5	37.5	-1.07
MHC_112_32222758	32222758	32222783	8295	ATG GCA ACC AAT TAC CCA GAT CCC A	48	58.2	25	-5.4	26.7	-0.08
MHC_113_32231053	32231053	32231078	7737	GTACCTGAGCCCTTCCTTTCTGACT	52	57.7	25	-3.6	17.2	0.5
MHC_114_32238790	32238790	32238815	8005	CTGTGACCACCTCATTGCTCCCATA	52	58.3	25	-3.5	26.4	-0.05
MHC_115r_32246795	32246795	32246820	8272	AAACTACACCTCGTCGTAATCTGCG	48	58.2	25	-3.6	28.6	-0.34
MHC_116_32255067	32255067	32255092	7704	AAGGAGAGGGTTGAGTGTGTTGAGG	52	58.1	25	-1.9	-250	2.4
MHC_117_32262771	32262771	32262796	10258	ATTCCAACACACAGAAAGAGCAGGC	48	58	25	-3.5	13.4	0.79
MHC_118r_32273029	32273029	32273054	5994	GTT AGC ACA CAC TGA GGT CCC TAC A	52	58	25	-4.6	20.5	0.27
MHC_119_32279023	32279023	32279048	7417	GGGAACAGGATACAGGAGGAGGACT	56	58.5	25	-1.6	11.7	0.51
MHC_120_32286440	32286440	32286465	8091	TCACTACTGTCTCTCCCATCCAGCC	56	59	25	-3.6	26.2	-0.09
MHC_121r_32294531	32294531	32294556	8535	CTT TCT AAA CTG AGC GGC ACC CAA A	48	58	25	-3.9	16.8	0.53
MHC_122r_32303066	32303066	32303091	4006	CAG GAT AAA GCA AGA AGT GGG CAG G	52	57.9	25	-3.1	25	0.01
MHC_123_32307072	32307072	32307097	9868	ATAAGGGGTACACAGGTTGGGTCTC	52	57.5	25	-3.6	25	0.04
MHC_124b_32316940	32316940	32316965	9596	CCCTGCATTACTCTTGACCACCAGA	52	58	25	-7	25	0.04
MHC_125b_32326898	32326536	32326561	3759	GCCCAGGTAGAGAGTGAGAGTGAAA	52	57.5	25	-3.1	7.8	1
MHC_126r_32330295	32330295	32330320	12314	AAA GCG GAG CTG ATG AAA GTG GAA C	48	58	25	-6.3	26.9	-0.13
MHC_127_32342609	32342609	32342634	2131	AGAGAAGAAACTGCCTTGTGATTGC	44	56.1	25	-3.5	19.2	0.44
MHC_128_32344740	32344740	32344765	15887	GGCAAAGCACCTCATTAAACTTGGC	48	58	25	-4.8	28.1	-0.22
MHC_129_32360627	32360627	32360652	5820	GCTGGATTCTATACACACAGAACAA	40	53.2	25	-5.1	33.9	-0.82
MHC_130_32366447	32366447	32366472	7915	TAACATAGGCTTCCATACCTGCGGC	52	58.7	25	-4.7	36.2	-0.93
MHC_131_32374362	32374362	32374387	8174	TTTCAACTTCATCTCCCTGCCTTCC	48	57	25	-3.1	-1.3	1.45
MHC_132_32382536	32382536	32382561	7706	ACCTCCTGCCATCCTCAGTTTATCT	48	57	25	-3.6	26.1	-0.11
MHC_133_32390242	32390242	32390267	8515	GCTTCATTTCTGGGTCTCAACCACT	48	57.2	25	-5	43.7	-1.73
MHC_134_32398757	32398757	32398782	7501	GGCAAGCAGGGTAATAAGGAAGCAA	48	57.6	25	-3.1	27.2	-0.24
MHC_135_32406258	32406258	32406283	8157	TAGGGCATTTGAGACAGGGAACAGG	52	58.2	25	-3.1	0.6	1.48
MHC_136b_32414415	32414415	32414440	8478	GCCACCATCAAGCCTCTTTCTTTGA	48	57.7	25	-5.5	27.7	-0.15
MHC_137_32422894	32422894	32422918	7984	AACCATGTCTCTTACAGCCCTCTT	45.8	55.5	24	-5.4	31.2	-0.49
MHC_138_32430877	32430877	32430902	9751	ACCTAGAGAGTTGGTTGATGGTGCT	48	57.4	25	-4.4	25	-0.03
MHC_139_32440629	32440629	32440653	6142	GTTTGTGCCTACTTCACAGGTGGG	54.2	57.9	24	-5.2	33.9	-0.9
MHC_140_32446770	32446770	32446795	7515	TAATAACACACCTCCAACCAGGGCA	48	57.9	25	-4.7	33.1	-0.72
MHC_141_32454285	32454285	32454310	8184	ACACCAATGTTTATCTATTCCCTGG	40	52.8	25	-5	25	-0.03
MHC_142_32462469	32462469	32462494	8098	GACCCCATCCTGAAAACCTTACCCT	52	57.9	25	-2	9.2	0.67
MHC_143_32470567	32470567	32470592	8466	ACAGCGGAGATGTTTGTGACCCTTA	48	58.2	25	-3.6	22	0.23
MHC_144b_32479033	32479033	32479058	8008	GAGATCCCAGTGACGTTGCTCACAG	56	59	25	-3.1	28.9	-0.22
MHC_145_32487041	32487041	32487066	7841	GTAACAGGTGGCAGCATTTCCCTAA	48	57.3	25	-4.7	19.8	0.3
MHC_146_32494882	32494882	32494907	8260	TGGTAAATCAAACGGAGAGGAGGCA	48	57.9	25	-3.6	14.2	0.65
MHC_147_32503142	32503142	32503167	7555	GCCAGGAGTGTGGGTTTGTTTAGTG	52	58.2	25	-5	35.4	-0.86
MHC_148b_32510697	32510697	32510722	7599	CTGAAGAGCCTACCAAACCTTGCAC	56	59.3	25	-6.3	35.9	-0.75
MHC_149_32518296	32518296	32518321	4043	TGGATATGGCAAAGAAGGAGACGGT	48	57.8	25	-3.9	-354.5	1.37
MHC_150_32522339	32522339	32522364	12827	AGACAAGCATGGATTAAGAAAGCAA	36	53.5	25	-5.4	27.7	-0.24
MHC_151_32535167	32535167	32535191	7777	GGACAGTTTGTGACTAGCCTCCTC	54.2	56.7	24	-4.6	33.3	-0.54
MHC_152_32542943	32542943	32542968	7959	GGCTCAAGCTCCTACACCCTTTCAT	52	58	25	-6.3	38.8	-1.1
MHC_153_32550902	32550902	32550927	7778	TCACATGCTACCACATAGGCACTCA	48	57.8	25	-5.4	36.2	-0.85
MHC_154_32558680	32558680	32558705	6697	GGA CAC ACT CGG CAT GTA ATT TGC T	48	58	25	-5.3	35.4	-1.01

MHC_155_32565377	32565377	32565402	13682	CAGAGAATGCCGTCAAATGCAGACA	48	58.1	25	**-7**	32.3	-0.73
MHC_156_32579063	32579063	32579084	8610	tgcttttcctcCTCTGCTTCAG	50	57.2	22	-3.55	26.2	-0.09
MHC_157b_32587669	32587669	32587694	3272	CCTCACAATCTCCCTTTCTCAGGCA	52	58.2	25	-4.7	29.4	-0.21
MHC_158_32590941	32590941	32590966	7453	CCATCTTACAGAGCTAGTCATTCCT	44	53.7	25	-6.3	23.4	0.11
MHC_159_32598394	32598394	32598419	7539	AAACACGGGAAAACAGACCTCTCCA	48	58.3	25	-4.6	30.6	-0.46
MHC_160_32605933	32605933	32605958	7576	GAG GTT CCT ACA TGG CAA AGC TGA C	52	58	25	-6.3	30.2	-0.26
MHC_161_32613508	32613508	32613534	13669	GCAAAATAAGCACAAACCAAGATGGC	42.3	56.6	26	-5	37.1	-0.83
MHC_162_32627179	32627179	32627203	3512	GTGCATCAGGCCCACTCTACAAAG	54.2	57.8	24	**-9.3**	38.3	-0.85
MHC_163_32630690	32630690	32630715	4226	TGGGTCTTTGCAGGATACACAGTCA	48	58	25	**-7**	19.3	0.37
MHC_164_32634916	32634916	32634941	12063	AAACCACGATCCCATGAATGTCCAC	48	57.6	25	-5.3	22.3	0.18
MHC_165_32646979	32646979	32647004	6158	CTCTCTTTCCATAAACCTGATGTCT	40	52.4	25	-3.9	19.6	0.52
MHC_166b_32653138	32653138	32653162	7826	CCCTACCCGGAAATCAATCTCTGCT	52	60	25	-9	35.5	-0.59
MHC_167b_32660963	32660963	32660988	11945	AACTAGCAGCCCTTGCATTTCTGTC	48	57.8	25	**-7**	17.1	0.52
MHC_168_32672908	32672908	32672933	5754	TGACATCTCTTCCTACCCTGTGTGC	52	58	25	-3.3	25	-0.02
MHC_169_32678662	32678662	32678687	7800	CTCAAGAGGTCCCGAAGCAAGTAAT	48	56.6	25	-3.6	25	-0.02
MHC_170_32686461	32686461	32686487	7727	TGAGATCAGCAGGGTATCTAAAGTAA	38.5	53.1	26	-4.6	29.8	-0.36
MHC_171_32694189	32694189	32694214	8945	TTCCTGGTCTCGCTCTTCTTCAGTC	52	58	25	-3.6	24	0.07
MHC_172b_32703134	32703134	32703159	7337	AGTGCAAACACATTAAGATGCTGCT	40	56	25	**-7**	33.1	-0.85
MHC_173_32710472	32710472	32710496	9203	GACTCTCACCCATCACTCCTCACA	54.2	57.4	24	-1.6	-0.2	1.13
MHC_174_32719674	32719674	32719699	8776	AGTCTGCCTCACCTCCTGAATGAAG	52	58	25	-3.5	21.1	0.22
MHC_175b_32728450	32728450	32728475	6066	AGTGGAAGCATAAAGTGGAGGGGAA	48	57.6	25	-3.1	20.1	0.37
MHC_176_32734516	32734516	32734541	7452	GCTCCACAAGAAACAAACTGCCCCT	52	59.8	25	-3.1	26.7	-0.14
MHC_177_32741968	32741968	32741993	3569	CATGCTCACTTTGTCCTATCGCTGG	52	58	25	-5.38	18.7	0.47
MHC_178_32745537	32745537	32745562	15593	CAACACACCTCTGCCTGTCTCTACT	52	58	25	**-3.3**	22.2	0.24
MHC_179_32761130	32761130	32761155	5108	GCCCGCCTTTCTCCTATCCTTTAGA	52	57.9	25	-3.6	28.8	-0.33
MHC_131_32766238	32766238	32766263	10118	GGAGATGGGAAGGGTGAGAGAATGT	52	57.5	25	-1.5	-32.6	2.35
MHC_132r_32776356	32776356	32776381	6175	GGT CTG GAT TTG AGG GGT CAG GAT T	52	58	25	-3.6	29.5	-0.4
MHC_133_32782531	32782531	32782556	9759	TATGGTAGGCAAGTGTTTGGTGGGT	48	58	25	-3.9	32.6	-0.7
MHC_134_32792290	32792290	32792315	6433	GCATGTGAAGAAGAGTCCACCTTGG	52	57.8	25	-5.4	30.1	-0.32
MHC_135_32798723	32798723	32798748	8091	GCCTTCCTGTATATTTCTGCTTCCA	44	54.9	25	-3.9	7.3	1.1
MHC_136r_32806814	32806814	32806839	7657	GGC TGA TGT CTT GGA GAT GGT CTG T	52	58.3	25	-3.2	17.2	0.44
MHC_137_32814474	32814474	32814496	7782	GAATCCACACAGAAGGAGACACC	52	57	23	-4.6	30.1	-0.31
MHC_138_32822253	32822253	32822278	8358	AAGTTGTAGGCGAATTGGGAAGTGG	48	57.5	25	-5.4	1.9	0.96
MHC_139r_32830611	32830611	32830636	7946	ACC TGT TCA TTC CTG ATG ACA CCC T	48	57.6	25	-5	38.4	-1.09
MHC_140_32838557	32838557	32838582	9632	TTCAGTCCACCATCAACTCAGGTCC	52	58.2	25	-4.4	28.5	-0.16
MHC_141_32848189	32848189	32848214	4076	CAAACCATACAAATCCTCCCTGCCC	52	57.8	25	-3.1	0.9	1.36
MHC_142_32852265	32852265	32852290	10411	TAGAGACAGGGAAGCACAGGGATCT	52	58.2	25	-4.6	11.1	1.02
MHC_143_32862676	32862676	32862701	8271	TCTTGGCACTATGAGACACTGGGAT	48	57.2	25	-3.2	20.9	0.18
MHC_144r_32870947	32870947	32870972	5993	CTC TGG GCT ATG AAC TTG CTA CTG C	52	57.5	25	-3.1	32.6	-0.52
MHC_145_32876940	32876940	32876965	11776	ATCGCAAATGCTGACATCCAAGGAA	44	57.6	25	-5.1	36.1	-0.89
MHC_146_32888716	32888716	32888741	6873	GGTCTCAGGGGAGTTTCTGGACAAT	52	58	25	-3.5	35.1	-0.95
MHC_147_32895589	32895589	32895614	7392	CTCCCCTACCAAGTCAACCAAGACA	52	58	25	-2.9	37.9	-1.23
MHC_148_32902981	32902981	32903006	8088	AAGGTAAGTCCCTCCCAACAACCAG	52	58.1	25	-4.7	32.5	-0.68
MHC_149_32911069	32911069	32911094	7462	TTACTCATCAGGGTGGTATCCGAGC	52	57.7	25	-3.6	22.6	0.17
MHC_150_32918531	32918531	32918556	7916	TAAGGCACCTGGAAGAAGATGGAGC	52	58.3	25	-4.7	30.6	-0.39
MHC_151_32926447	32926447	32926472	7621	ACTCAGAACGGGTTGGGGATCAAAT	48	58.2	25	-4.6	23.7	0.06
MHC_152_32934068	32934068	32934093	8912	AGTGGGAAGGAAGGGCTTGATGATT	48	58	25	-3.5	27.8	-0.18
MHC_153_32942980	32942980	32943005	9651	GGCAAAGGTGACAGAAGAAGCAACT	48	57.9	25	-3.1	26.7	-0.13
MHC_154_32952631	32952631	32952656	6237	CTACCTAAAATCCTACCGAGCACGC	52	57.3	25	-3.6	33.7	-0.57
MHC_155_32958868	32958868	32958893	7778	CAATGTTGCTCTCACTCACCTTGGC	52	58.6	25	-3.9	17.9	0.52
MHC_156_32966645	32966645	32966671	6172	TTCTTCACATACAAGGGGTCCTGACA	46.2	57.4	26	-4.7	32.6	-0.59

MHC_157r_32972818	32972818	32972843	11264	GTA GAC CTG ACA TTG GGC ACT GAG A	52	58	25	-3.1	19.1	0.45
MHC_158_32984082	32984082	32984107	6713	CTGGTGAGGGAAAAGAAAGGTGGAC	52	57.4	25	-1.6	-1.7	1.31
MHC_159_32990795	32990795	32990820	3685	ATCCACACCTATGCTCGTTATATTG	40	52.9	25	-3.9	19.9	0.25
MHC_160_32994480	32994480	32994505	12331	TTGGGCAACAGTCTCAATATGTAAC	40	53.6	25	-3.9	21.7	0.17
MHC_161_33006811	33006811	33006836	7922	AGAAGTGAGTGTCCTCCCAAACTCC	52	57.9	25	-4.5	36	-1.18
MHC_162_33014733	33014733	33014758	7755	GGGAAGGGAGAACAGGTCAATGTCT	52	57.9	25	-3.3	25	-0.03
MHC_163_33022488	33022488	33022513	8335	GGAGTTGGACCTATGGAGTGGGTTA	52	57.2	25	-4.4	30.7	-0.43
MHC_164_33030823	33030823	33030848	5815	AGTCTCCCATTCTTCCTGCTGCTAC	52	58.1	25	-3.1	-5.3	1.77
MHC_165_33036638	33036638	33036663	10298	TCAGAGAGCAAACCTAGCCATCCAG	52	58.1	25	-4.2	23.4	0.09
MHC_166_33046936	33046936	33046961	8547	TGGAGTTTGAGGAAGAGGTGAGGTG	52	57.9	25	-1.9	0.3	1.64
MHC_167_33055483	33055483	33055508	7079	CCACTCTGGGCAGGAAATAGGATCA	52	58	25	-5	28.4	-0.18
MHC_168_33062562	33062562	33062587	6836	GCTTTCCAACCAACCCCAAACTTCA	48	58.3	25	-3.9	-49.5	2.33
MHC_169_33069398	33069398	33069423	9042	TGCACGAAGAGAAACCAGGCTAAGT	48	58	25	-7	22.4	0.19
MHC_170_33078439	33078439	33078465	8407	GGTCTTCATCTTGTAGGTAATGGAGC	46.2	55.2	26	-3.4	20.5	0.4
MHC_171_33086847	33086847	33086872	8002	TCTATGGCTTGGAGTGTGGTGAACT	48	57.6	25	-3.1	25	-0.01
MHC_172_33094849	33094849	33094874	8013	AGAAATAGCCTTCCATGAGGTTGCG	48	57.4	25	-5.4	34.5	-0.89
MHC_173_33102862	33102862	33102887	10187	ACAGTTGACATTGGGGTACTCAGAA	44	55.8	25	-3.6	17.2	0.29
MHC_174_33113049	33113049	33113074	1249	GCAGCCCCAGATACCATTCTTTGTC	52	58	25	-3.1	19.1	0.45
MHC_175_33114298	33114298	33114323	9097	ACTACTTCTCTGCCTCTCACTGGGT	52	58.4	25	-3.1	22.3	0.17
MHC_176_33126206	33123399	33123420	10423	ccaccatgattcTGTCCCCTAT	50	56	22	-5.3	17.4	0.47
MHC_177_33133818	33133818	33133843	10837	AAATAAAGCAACACACCCCTGCCTG	48	58	25	-5	32.2	-0.55
MHC_178_33144655	33144655	33144680	6434	TGGATGTTAGGACGAGGAGAGGACT	52	58	25	-3.6	11.5	0.63
MHC_179_33151089	33151089	33151114	7820	TACATGGTAGGGTAGGTGGTGTGTC	52	57.2	25	-5.4	23.9	0.09
MHC_180_33158909	33158909	33158934	5795	TCACATTGAGGGAAGAGGCTGACAA	48	57.9	25	-3.9	17.5	0.6
MHC_181_33164704	33164704	33164729	6852	ACAGTTCCCTAACGAGAAGGTGGTC	52	58	25	-4.7	28.3	-0.35
MHC_182_33171557	33171557	33171581	11422	GATTCCCTGCCTTCCATGAGCAAC	54.2	58.1	24	-5.4	30.3	-0.39
MHC_183_33182978	33182978	33183003	8150	GGAGAAGTGTGTGATGTCGCTGAAC	52	58.1	25	-3.6	21.4	0.33
MHC_184_33191128	33191128	33191153	9851	AGTCCTGGGATCTTGCCTTAGAGAT	48	56.5	25	-4.6	27.3	-0.17
MHC_185_33200979	33200979	33201004	5604	ACATGGGTTTAGGAAAGTTGCTGGC	48	58	25	-5.4	14.6	0.72
MHC_186_33206583	33206583	33206608	9897	GACCACAGCATACAGACCATCCAGT	52	58.3	25	-3.1	7.4	1.02
MHC_187_33216480	33216480	33216505	5387	TGGAAGAGGAAGTGAGGCGAAATGA	48	57.9	25	-3.6	8.3	0.57
MHC_188_33221866	33221866	33221892	8341	ACCAGATACCAATGCCAGAGGAAGAG	50	58	26	-3.14	33	-0.49
MHC_189_33230208	33230208	33230233	7998	CAAAGTGGTGGCAGGCATTTCTACA	48	58	25	-3.9	16	0.54
MHC_190r_33238206	33238206	33238231	8088	GTC CCT ATG GCT CCA GTT TCT GAC A	52	58	25	-5	35.3	-0.72
MHC_191_33246294	33246294	33246319	8305	AGTAGGGGTCACACTCACCATAGGA	52	57.9	25	-4.4	31.9	-0.59
MHC_192r_33254599	33254599	33254624	7705	GCT CAG TTG TTT CTT GGG GAT GAC C	52	58	25	-3.5	19.7	0.28
MHC_193_33262304	33262304	33262329	8018	CAACTAAGGAGGAGAGATGCCTGGG	56	58.1	25	-4.7	31.2	-0.44
MHC_194_33270322	33270322	33270347	8291	ACTTCTCACCCCATCAAGGTTCTGG	52	58.2	25	-5	32.8	-0.5
MHC_195r_33278613	33278613	33278638	8278	TCA GAG GCT TCC ATC ACC ACT TGT T	48	58.1	25	-3.1	34.6	-0.59
MHC_196_33286891	33286891	33286916	7498	CCTTAACCACTTGCTTCTACAGGGC	52	57.6	25	-4.9	22.2	0.19
MHC_197r_33294389	33294389	33294414	8944	AAG ATG GAG GAA GCC TGG GAT GAA G	52	58	25	-4.7	26.4	-0.09
MHC_198_33303333	33303333	33303358	6845	AAGGAGAGAAATGGGATGGGCGTTA	48	57.7	25	-3.6	2.4	1.53
MHC_199_33310178	33310178	33310203	8780	TCAGGATCAAATCAAGGTACATGCG	44	55.5	25	-5.4	30.3	-0.39
MHC_200r_33318958	33318958	33318983	7290	TCT GAA TGT AAA GCT GGG AGA GGC A	48	57.7	25	-6.3	26.3	-0.08
MHC_201_33326248	33326248	33326273	8431	TGAGTGAACCAGAGATTGAGGCAGT	48	57.6	25	-3.1	26.7	-0.12
MHC_202_33334678	33334678	33334704	7562	ATTGTATAGTTGTGGTGACGCTCCTC	46.2	56.8	26	-3.6	34.1	-0.7
MHC_203_33342241	33342241	33342266	9629	ATGACCCCACTATGCTGCTGATGAA	48	58	25	-3.1	8	0.84
MHC_204_33351870	33351870	33351895	6548	ACAGAATTGGGCATAGGAGGTCAGG	52	58	25	-5.3	25	-0.03
MHC_205_33358418	33358418	33358443	7818	ATGGGCTGAAGATCACAAGGACCAG	52	58.5	25	-5	27.2	-0.21
MHC_206_33366236	33366236	33366261	8868	ACCCTGTGATGCAAGTGAACCATTG	48	58	25	-7	20.2	0.39
MHC_207_33375104	33375104	33375129	6866	CTCCCTACCACAACCCACGAATGTA	52	58	25	-3.6	21.8	0.15

MHC_208_33381970	33381970	33381995	8431	AGCATACCCCAAGTCAACAGCATCT	48	58	25	-3.1	26.3	-0.05
MHC_209_33390401	33390401	33390426	8732	ACACAAACACAGACTTCAGGCACAG	48	57.9	25	-3.1	2.7	1.02
MHC_210_33399133	33399133	33399158	6997	GTTTCAGGGAAGGGCAGAAGTCTCT	52	58.2	25	-3.5	15.2	0.51
MHC_211r_33406130	33406130	33406155	9294	TTC AGT CCT TGC TCC TAC AAC CTC C	52	58	25	-3.9	26.8	-0.07
MHC_212_33415424	33415424	33415449	5145	AGACTGAAAATGGGGATGAGGGTGT	48	58	25	**-1.6**	1.9	1.24
MHC_213_33420569	33420569	33420594	12500	CAGCCAGAGGAAACCAAAACCACTT	48	57.8	25	-3.1	27.5	-0.2
MHC_214_33433069	33433069	33433094	5534	ACCCAATGTAGAGAAACGACGGTGA	48	58	25	-4.4	14.3	0.43
MHC_215_33438603	33438603	33438628	5631	CCAATCTCCAGAAATCGCTGCTGTC	52	58.2	25	-3.6	27.5	-0.21
MHC_216_33444234	33444234	33444259	11946	GGAAAGACAATCCCTCAAAGAGAAA	40	53.2	25	-4.6	32.5	-0.53
MHC_217_33456180	33456180	33456205	6650	TGCTTATACCCTGCACACCCTAAGT	48	57.5	25	-7	32.1	-0.6
MHC_218r_33462829	33462829	33462855	9516	TGT TGG TAA GCC ACT GTT TAT GAC CT	42.3	56.5	26	-5	37	-0.89
MHC_219_33472346	33472346	33472371	6755	TGTCATCTGGTTCGCTAAGGTGGAT	48	57.8	25	-3.6	17.2	0.62
MHC_220_33479101	33479101	33479126	8456	TGTTCCACAGCTATTGCCACAGGTA	48	58.2	25	-6.3	35.1	-0.68
MHC_221_33487557	33487557	33487582	7349	AGGTGTCTCAGGGATTTGGAGATGG	52	58	25	-4.7	32.3	-0.64
MHC_222_33494906	33494906	33494931	7927	TCAAGGTCTGAGGTCATTGGTCTGG	52	57.9	25	-3.9	23.1	0.11
MHC_223_33502833	33502833	33502858	8620	ACATCTCTGTGAGGCAAAGTCAGGT	48	57.9	25	-3.6	29	-0.42
MHC_224_33511453	33511453	33511478	7356	GTTAAAGGAGGAGAGGCTTGGGGAA	52	58	25	-0.8	20.7	0.25
MHC_225_33518809	33518809	33518834	8371	GCTGAGTGTCAACAAGAGTGTGTCC	52	58	25	-3.5	34.9	-0.55
MHC_226r_33527180	33527180	33527205	7722	TAA CCC TTC TCT GGC TTC ACC TGT G	52	58.2	25	-3.3	36.9	-0.8
MHC_227_33534902	33534902	33534927	4228	GGGTCAACCTGTCTTGGATCTGCTA	52	58	25	-4.6	36.4	-0.77
MHC_228_33539129	33539129	33539155	10277	AGGAGAGTAACAGAAAAGGAGGGCAT	46.2	57.3	26	-3.1	-13.9	2.14
MHC_229_33549407	33549407	33549432	6134	ACGGGAGAGGAGTTGAGGAGGTAAA	52	58.4	25	-3.6	-9.9	1.46
MHC_230r_33555541	33555541	33555566	10775	TTC TGT GTC AGT TCG CCA CAT TAC A	44	57	25	-5.2	33.1	-0.77
MHC_231r_33566316	33566316	33566341	8457	CAC CAT CGG CAT ACA CTC ATT GCT G	52	58.5	25	-5.1	35.4	-1.01
MHC_232_33574773	33574773	33574798	8022	TAGTTCAAGTCACCTTCCATGTCAA	40	54.1	25	-5.4	16	0.46
MHC_233r_33582795	33582795	33582820	8082	GAG AAT CCA TGA AAG AGT GGT TTG T	40	53.6	25	-5.4	30.3	-0.52
MHC_234_33590877	33590877	33590902	7007	TCTGCTACACTAACCTTGCTCCTGT	48	57.4	25	-3.3	26.2	-0.09
MHC_235_33597884	33597884	33597909	6809	ACACGAGTACATCCTCACACACCTC	52	58	25	-3.6	29.3	-0.43
MHC_236_33604693	33604693	33604718	9181	GCCAAATCTGACCTTTCTTCACGCT	48	58	25	-3.9	19.5	0.34
MHC_237_33613874	33613874	33613899	8348	AACGGTGTGGAGGAGAACTTGACAT	48	58.1	25	-3.6	11.7	0.51
MHC_238r_33622222	33622222	33622247	9585	GGT CGT TCT CAG GGT TCT TGT TGT T	48	57.4	25	-3.6	15.3	0.62
MHC_239_33631810	33631810	33631832	8901	CCA TGC CAG TCC TGT TTC CTG TT	52	59.7	23	-5.3	26.3	-0.07
MHC_240_33640708	33640708	33640733	6035	TCAGAGATTGTGCCCTCAAGTCATA	44	55.5	25	-3.9	30.1	-0.53
MHC_241_33646743	33646743	33646768	7503	ACGAACCCAGACGTATTTGGTGGAA	48	58.4	25	-6.3	35.5	-0.8
MHC_242_33654246	33654246	33654271	8797	TCAGAGTGTGTTCTTTCTCAGGGCA	48	57.8	25	-3.2	32.7	-0.8
MHC_243_33663043	33663043	33663068	7799	TCCACTCCAAACACACATAGGCTCA	48	57.9	25	-3.1	9.9	0.8
MHC_244r_33670842	33670842	33670867	5569	CCT CTG CTC ATT CAG TCT AGT GCC A	52	58.1	25	-4.2	29.3	-0.43
MHC_245_33676411	33676411	33676436	10719	CGTTCCTTGTGACTGTGAAACCCAG	52	58.2	25	-3.6	26.2	-0.13
MHC_246r_33687130	33687130	33687155	7527	TCA GCC CTG TGA TCC TGT TGA GAA A	48	57.9	25	-4.6	26.3	-0.11
MHC_247_33694657	33694657	33694682	7849	TCTGTAGACTTCTGTGGGCAGTGAG	52	57.7	25	-3.6	37.8	-1.16
MHC_248r_33702507	33702507	33702531	8278	GAA TGC TAT CAG CGA GGG AAA GGC	54.2	58	24	-4.7	35.3	-0.83
MHC_249_33710784	33710784	33710809	8061	TCCATCACCCCAGTCAATGCTTTCT	48	58.1	25	-3.4	15.9	0.53
MHC_250_33718845	33718845	33718870	7164	AGTCCTGGCTTTCACTGTTTCCACT	48	58.3	25	-5	21	0.32
MHC_251_33726009	33726009	33726034		TAGAAGCATGGGATGTGTATGGCGA	48	58	25	-5.3	27.6	-0.23

Supplemental Table 1. 500 capture, hybridization oligonucleotides designed using Anotholigo. The genomic position (HG19) of the hybridization oligos as well as the sequence and spacing between primers is provided in addition to other design criteria metrics including Dg, Tm, and GC%.

**MHC-1**	Forward Primer<br>5'-GAAAGACAGAGCAAGACCAATG-3'<br>Reverse Primer<br>5'-CAGCTAGGGATGGCAAGAG-3'<br>Probe<br>5'-/56-FAM/AGGACAATGACAGACGGGAAGGAG/3IABLFQ/-3'
**MHC-2**	Forward Primer<br>5'-CCCTGCAAAACCTGTGTT-3'<br>Reverse Primer<br>5'-GTTCTTACTCCAAGGTCACG-3'<br>Probe (reverse complement)<br>5'-/56-FAM/TCACTTACACGGCTTCCCCATCC/3IABlk_FQ/-3'
**MHC-3**	Forward Primer<br>5'-TACCCCAAATCCCATCCA-3'<br>Reverse Primer<br>5'-CCCTGTAATTATCCCTGCCA-3'<br>Probe<br>5'-/56-FAM/TCTAGTGATTTCTCCCCAGTGCAAGC/3IABlk_FQ/-3'
**MHC-4**	Forward Primer<br>5'-GGCCTCTAGGTTTCTGTCTT-3'<br>Reverse Primer<br>5'-CAGATCACAATGGGAGAAGG-3'<br>Probe<br>5'-/56-FAM/AGGCAGGATCATTAGCAACATGGGA/3IABlk_FQ/-3'
**MHC-5**	Forward Primer<br>5'-CACAATTGACCCAGTTTGCAG-3'<br>Reverse Primer<br>5'-CCGATAACCTCTCCCTTGATTAC-3'<br>Probe<br>5'-/56-FAM/CCGACAGATGGCACTTCACAGAACA/3IABLFQ/-3'
**ACTB**	Forward Primer<br>5'-CAGCGGAACCGCTCATTGGCAATGG-3'<br>Reverse Primer<br>5'-TCACCCACACTGTGCCCATCTACGC-3'<br>Probe<br>5'-/56-FAM/ATGCCCTCCCCCATGCCATCC/3IABLFQ/-3'
**EGFR**	Forward Primer<br>5'-AGCATTGAAGAAAGTGCCAGTGTATTGATT-3'<br>Reverse Primer<br>5'-ATGGCCTCTGGCTTTGGATGTTG-3'<br>Probe<br>5'-/56-FAM/CATGGTTTGTGCTCTACCACCTGGTTACTGTGATTGC/3IABLFQ/-3'

**CLEC16A**	Forward Primer<br>5'-TCCAGCAGGTATTGTCCAC-3'<br>Reverse Primer<br>5'-CTGGAGGTCAGGGGTTTTGG-3'<br>Probe<br>5'-/56-FAM/ACATTTGGAGAACCCCGTGATCCC/3IABLFQ/-3'
**BRCA2**	Forward Primer<br>5'-AAATAATATCCTTAATGATCAGGGCAT-3'<br>Reverse Primer<br>5'-AGAACTAAGGGTGGGTGGTG-3'<br>Probe<br>5'-/56-FAM/AAAGTCTAGGAGCTGAGGTGGATCC/3IABLFQ/-3'
**CETP**	Forward Primer<br>5'-TGGATACCATCTGATAGCGG-3'<br>Reverse Primer<br>5'-CCGTGGACACACTAACAGG-3'<br>Probe<br>5'-/56-FAM/TGTCTTTCCATAAGCTGCTCCTGCA/3IABLFQ/-3'
**KCNJ2**	Forward Primer<br>5'-GAAGACGGTATGAAGTTGGC-3'<br>Reverse Primer<br>5'-AAGCCAGGCAGAAGATAACC-3'<br>Probe<br>5'-/56-FAM/AGTAAAGTCCACACCCGACAACAGT/3IABLFQ/-3'
**KCNE1**	Forward Primer<br>5'-TGTGGCAGGAGACAGTTCAG-3'<br>Reverse Primer<br>5'-CGATGTAGACGTTGAATGGG-3'<br>Probe<br>5'-/56-FAM/CTCTACGTCCTCATGGTACTGGGAT/3IABLFQ/-3'
**NOS3**	Forward Primer<br>5'-CCTCTCAAGAAGGGCCTG-3'<br>Reverse Primer<br>5'-GTGCCGATCTCAGTGCTC-3'<br>Probe<br>5'-/56-FAM/GTGTCCAACATGCTGCTGGAAA/3IABLFQ/-3'

Supplemental Table 2. List of qPCR primers and probe sequences designed to amplify regions within several genes (ACTB, EGFR, CLEC16A, BRCA2, CETP, KCNJ2, KCNE1, NOS3) as well as five MHC locations (MHC-1, MHC-2, MHC-3, MHC-4 and MHC-5 as indicated by arrows in Figure 5a).

	Primer Start Position		Primer Sequences	
**Variant Position (HG19)**	**Forward**	**Reverse**	**Forward 5'-3'**	**Reverse 5'-3'**
29642970	29642636	29643047	AGATCTTCAGCCAGCCAGG	TGC TCT CCT AGT GTA CCT CTG TC
29782813	29782654	29782899	ATCAGCACGCAATTCCCAACTCAC	ATG ACC ATG TGG CCT GCT TAA AC
29873342	29873156	29873489	ACT AGG TTC CCT CAT CCT CTT GCT	GCA TCT CCC ACC ATC AGT TCA GAC T
29881169	29881045	29881547	AAG AGG ATG CCA CCA GGG TTG	AGC CTC CTA TGC ATG CCC AG
30064186	30063819	30064228	GGG AGG TGG ACA CCT TGG AAA TAA	ATG AAT TAG TGG GAT TGG AGC ATG TG
30298379	30298174	30298511	TGTAGGTGGACAGAGGTGGA	GAG AGG TGA CAT TTG GGG TTG ACT G
30313566	30313343	30313805	CCA GAG ACG TGA GTG CTC CAA C	ACCACTGGCATGAGTAAGTGAGCA
30314841	30314757	30314968	CTCACTCAGCAGCTTGCATTTCCA	CTT TGA GAC TGG GAA AAG ACC TGA G
30523754	30523607	30523955	CAG TCC TCC CAG ACA CCC TAT TTG G	CGA GGA AGA AGC CAT TCA GCG T
30720263	30720127	30720333	CCAAAGCAGTTGCCATAGGCTCTT	ACT TGG TTC TGA GTC ACA CAT GGC
30889980	30889806	30890156	AAACGATAAGGAAGGGATGGCTGG	TTG GGA TCT CTG GCC TCC ATA CTT
30917212	30917008	30917284	TTGCCTCAGACAAGCTCCTGACAA	GTG TGG TAT TCT CAT TGG CTG TCA
30993958	30993858	30994181	CAG CAT CTA CTG CAG GTT CTG AGA	ACTAGAGACTGTGGTGTTCTCGGA
31097638	31097168	31097655	ACA ATG GGT TTC CCT CTG CAA	GAG CCC CTG ATG TTG TCT AGG GA
31122977	31122801	31123066	AAG TCT GGC TGA GAT CGT GGA ACA	TTT AAC TAG CAC CTG GCC TCA CC
31167845	31167697	31167938	CCA GTG CCC TTG GAG ACT AGT	GGA GCA AAG ATG CTA CAG CAG GT
31193053	31192946	31193148	GGC TGC TCT CCA CAT TCT CCT AAA	ACC CAA GAG CCT TCA GAC AGT CAA
31524014	31523855	31524122	GTA AAC CTC CCA TGC AAG CCT G	TGC TGG AAA GTA CTG TTT ACC TGG C
31634758	31634633	31634865	TGGATTTCCTGGTTCTGTGGGCT	TATATTGCTGGACGGTGGCAGACT
31648132	31648010	31648297	GAA CCC TTG GAG CCA CTT ACC A	GCC CTC AAG CAG GAT GTT ATG C
31648133	31648010	31648297	GAA CCC TTG GAG CCA CTT ACC A	GCC CTC AAG CAG GAT GTT ATG C
31694325	31694205	31694488	TTTCCCTCCTTCACACTATCTGCC	ATGACACTTCCAGGGACATTCA
31740352	31740241	31740668	TCT GTA TGC ACA CAG TAG AGG CTC	TTC AAG GTA AGG GAT AGA CTG GGC
31740356	31740241	31740668	TCT GTA TGC ACA CAG TAG AGG CTC	TTC AAG GTA AGG GAT AGA CTG GGC
31795949	31795855	31796002	CCTTCACGGACACCGAGCG	CTCCGTCGTTGATCACCTGGA
31833072	31832893	31833250	GCC TGG CAA TGC TGA GAG TG	AGC TAC ACT GCA AGC AAC CCT T
31870998	31870930	31871114	GAAGATGCCACTGTTTCCTGCCAA	ACCACACCCTCCCTATTGCATGAT
31994782	31961844	31962171	TGTAGGAAGAGGGTCCAGTGATCT	CCT GTA TGC TCA GGC TCC TAT G
32006858	32006739	32006925	ATCCAGGTCCCTGGAAGCTCT	TGAGCTTCTTGTGGGCTTTCCAGA
32054981	32054796	32055042	CTT GAC CTT AGG CAT CCA CAG GAT	AGA CCT CCA AGT CCT ACG CCT T

32312954	32312600	32313009	ACT GTT AAG GAA ACT TGG AAG ACC T	GGG AGC ACA TAG GGA AAC TAA GG
32360309	32359988	32360427	AACTGATCAGGCCAGGTGTG	TGA GAT CCG GCC TAT CAA TCA CAA T
32360327	32359988	32360427	AACTGATCAGGCCAGGTGTG	TGA GAT CCG GCC TAT CAA TCA CAA T
32559516	32559413	32559580	TGCAAGGATGCAAGGCTAGTAAACAG	TCCCTCATTCCTGGCTCCAATTA
32705528	32705421	32705954	ATGGCATGTCAATTAGTGCTTTCTAA	GCG AAC CTT GTT CTA TTT GCT TCT GT
32714960	32714786	32715143	CCA GTA ACA CAG GAG CAA CCA AGT	TCT CCT CAA GGT GAG AAG GGG A
32753753	32753641	32753888	ACTCCTGCTCCAGTCATGCTTCTT	AGG AAG ACA TTG ACA GCT GTA GGG
33039755	33039632	33039863	GGG CTA TTG CAC TTA GAA GAC CTG	TCT CCT GTT TAC CTG GAT CCT CCT
33081115	33080976	33081232	AGG ATG GGT TGA AAC CTG TCA GAA G	ATGCAGAAAGGTGGAAGTCAGTGG
33134364	33134182	33134498	GAG GAC TAT GCT TGT TAG GCT GGT	CCT GGG CAG AAG GGT GAG AT
33147589	33147320	33147642	CCAGCACTCTCCAAATTCACCCTT	AAC GCT GGA GAA AGC CTC TGG
33171247	33171122	33171375	AAG TCT GTG GAA AGG ACC TCT GAT C	ACT GCT CCT CCT TCA GTG AGA AG
33234563	33234360	33234703	TGA CAG CAT GGA ACA GGT CGT GT	TTGAGGTGGATGCAGGAGTAGCAA
33257396	33257271	33257511	GCA GAA CTC CAC TCC CTA AAC ATG T	GCA CTC TTG AGA GGT AGC CCT G
33269394	33269008	33269528	TCC TAG AGC TCC AAG AAG GTG AAT	AAG CAG AGT GAG GGC ACT CA
33280795	33280654	33281009	GCA TCA CCT GAC AAG TCT CTC AAG G	CTC AGC AGG AGC CTG TTC TCA
33289931	33289789	33290034	CAG CAT AGC CCC ATC CCT TCA	GGG AGG AAG AAT CAA CTT GCC AAC T
33383344	33383210	33383540	ACTTCTTGGCCTAGACCGACTTCT	GAAATGGGCAGAAATGGAGAGCAC
33457013	33456958	33457248	GGGTGGCCTCCAGAGGTTCC	TTA GTC AGG CAT GAT GGC ACG TAC CTG
33457926	33456996	33458165	AGT GTC ACT GTT AGT GAT GTT T	CAG TCT TTA ACA GTG CTT TGA TA
33481405	33481165	33481506	TTT GAG TGT GCA GCA GCC AG	GGA TGA GGC TGT GCT ACA CCT
33505847	33505733	33506003	GCG GGA AAA GAT TGC AAG GTA GTG	TCAGGAAGCTGTGTCATAGCCTGT
33556608	33556401	33556721	CCG AGC CTT TGG AGA ATG CTG G	CAG AAA TGG GTG AAG GAA GAT GCC
33563140	33563034	33563397	GAG AGT GGC CTG GTA AGA TGT ACA G	CCA TCC AGC AGG CGA TTC TCT
33583039	33582708	33583121	GAT CCT TTC ACC AGT CTC TGG CC	CTG TGT GGA ATT GAA CTG TCA GGG A
33583113	33582818	33583116	TAGTGGGTGCTGGGAGATACCC	GTG TGG AAT TGA ACT GTC AGG GAG A
33584003	33583949	33584268	TTC CTG ACT TGG TCT GGA GAC ACA	CGG AAA AGC CCT GCT CTA GAG T
33586057	33585876	33586121	GCTAGAGGAAAGATACAGGCACTCA	TGT GAA GGA AGT GTG AGC CAT CA
33586748	33586700	33586975	TGAGGAGGCATAGGAAGGCTTCAA	AAAGCACATGGCCTTGTTTCTCCC
33587065	33587007	33587336	GAGACCCAAAGTGGAGTGACCTC	GAC AGT TTC ACT TGC TCC CAG GAG
33587075	33586928	33587394	TGGTGGAGAGACAGAGGCTCTAAG	CTG AGC CAC AGC GAT GGG T
33587322	33587028	33587343	TCC ATT CCT GGC TGC TAC ACC	GGG ACA GTT TCA CTT GCT CCC A
33587824	33587399	33587838	CCA TCG CTG TGG CTC AGT TG	AGG GAA GGA CAC CTC CAG GAT

33590061	33589896	33590163	TATTACCGATCCCGTGGTGCCTTT	AGGGACGTGAACTGATTCCCTGTT
33590291	33590254	33590505	TCAGAGGAGCAGCTGGATGTGATT	GTC CAG CAT CAG TCC TGA GCT
33590471	33590254	33590505	TCAGAGGAGCAGCTGGATGTGATT	GTC CAG CAT CAG TCC TGA GCT
33592848	33592669	33592882	AGAGCCTCCTGGAGAATTAGAGGT	TGTTGGCCCTAATCAGCTACTCCA
33592881	33592753	33592964	TCC AGG AGC GGT TGA GAC TG	CTG ATG GGT CCA CCC TGT GT
33593577	33593401	33593691	ACATTTGTCAAAGTGCCTCTGCCC	CCACCCAACAAGTTGGTGATGGAA
33595525	33595496	33595687	CAG AAC TTG GTG CCA CAG AGG	AGTCTCAAGGTGGATGTGTGGCA
33595599	33595491	33595775	AGGAACAGAACTTGGTGCCACAGA	AGG GCA GAG TGT AAT CAA GTG GCT
33596499	33596353	33596806	TTCACAACACAGTGAGTCCAGGGT	AGGGCCACCCAATGTAAGTGATCT
33596868	33596688	33597091	AGCCCTCTTCTCCAGTATGAGTCT	TCT GAC TAT GGA TTG GGC CCT G
33598456	33598285	33598647	TCTTCCCAGCTTTCCCTTTGGTCT	TACCAAGAATCCACCCTGTGCCAA
33598613	33598525	33598750	ACTATTTGGGTTCTCTGCGTGTGC	AGAAACCCAGATTCACTGGTTGGG
33598642	33598525	33598801	ACTATTTGGGTTCTCTGCGTGTGC	GCCCTTTCTCCAGATAGCCAAAGT
33600895	33600717	33601042	GGTTGTGTCTGTGCCATTCTCTGA	AAGACCTGTTCCCACCCTTTACCA
33607224	33607181	33607412	ACCTTGTTAACCAACCCAGGGAGA	TGTCAATGCCACTCCAGCCTCTAA
33608379	33608319	33608507	AAGAAGTTCCGTGGTAAGACCTCC	AATGAAGGGAATGGCACGTCTGTG
33609965	33609862	33610089	GAGTAATCAATGCATGCACAGGGC	TCAACGCCCTCCCATTTCTGACT
33610350	33610244	33610487	AAGCTTTGCATGTGTGTCGAGGTG	GCCTCATTCACAGGCTTTGGGAAA
33610705	33610555	33610838	TGGGTCCATCCAGCAAAGTGATGT	CTTGAGGCCAAGCTCCATCTTTGT
33617403	33617324	33617507	AGACTGAATGACCTTTCCATCCTC	TGCTGCTCAATCAAGCACCAGAGT
33617464	33617407	33617877	AGATGATTGGAAGCAGATGCAGGC	CCACACAGGAGTTGCAGCATCAAA
33618162	33617952	33618258	TGTGGTTTAGTGACAGGGAAGGCT	ATGAGACACACTGCCCTGAAGCAT

Table 3. PCR primer location and sequences designed to validate high-confidence variant calls from NGS results.

Region	HG19 Position	Reference Sequence	NGS	Sanger	Depth of coverage	Tm °C	Notes
Intron	29642970	T	T/C	T/C ? POSSIBLE A	80	57	Sanger sequencing agrees with NGS
Intergenic	29782813	C	C/T	C/T	149	57	Sanger sequencing agrees with NGS
Intergenic	29873342	A	A/C	A/G ?	203	53	Sanger sequencing agrees with NGS (ref seq errors)
Intergenic	29881169	T	T/G	T	108	53	Sanger agrees with reference sequence
Intergenic	30064186	G	G/A	G	158	53	Sanger agrees with reference sequence
Intron	30298379	A	A/G	A	204	53	Sanger agrees with reference sequence
Intron	30313566	C	C/T	C	55	53	Sanger agrees with reference sequence
Intergenic	30314841	G	G/C	G	93	53	Sanger agrees with reference sequence
Intron	30523754	A	A/G	A	60	53	Sanger agrees with reference sequence
Intergenic	30720263	C	C/T	C/T	107	57	Sanger sequencing agrees with NGS
Exon	30889980	T	T/C	T/C	165	57	Sanger sequencing agrees with NGS
Exon	30917212	T	T/C	T/C	158	57	Sanger sequencing agrees with NGS
Exon	30993958	A	A/G	A/G	110	57	Sanger sequencing agrees with NGS
Intron	31097638	A	A/G	A/G	159	57	Sanger sequencing agrees with NGS
Intron	31122977	C	C/T	C	38	57	Sanger agrees with reference sequence
Intron	31167845	A	A/C	A	45	53	Sanger agrees with reference sequence
Intergenic	31193053	A	A/T	A	389	53	Sanger agrees with reference sequence
Intron	31524014	A	A/C	A	77	53	Sanger agrees with reference sequence
Intron	31634758	G	G/A	G/A	232	57	Sanger sequencing agrees with NGS
Exon	31648132	G	G/C	G	176	53	Sanger agrees with reference sequence
Exon	31648133	G	G/C	G	183	53	Sanger agrees with reference sequence
Exon	31694325	G	G/A	G/A	235	53	Sanger sequencing agrees with NGS
Intron	31740352	C	C/T	C	92	53	Sanger agrees with reference sequence
Intron	31740356	C	C/T	C	88	53	Sanger agrees with reference sequence
Exon	31795949	T	C?	Y?	33	57	Segmental duplication (software problems)
Intron	31833072	C	C/G	C	145	53	Sanger agrees with reference sequence
Intron	31870998	A	A/T	A/T	43	57	Sanger sequencing agrees with NGS
Exon	31994782	C	C/T	Y	122	53	Segmental duplication (software problems)
Intron	32006858	C	C/A	C/A	64	57	Sanger sequencing agrees with NGS
Intron	32054981	T	T/G	T	91	53	Sanger agrees with reference sequence
Intron	32312954	C	C/T	C	77	53	Sanger agrees with reference sequence
Intron	32360309	G	G/A	G	60	53	Sanger agrees with reference sequence
Intron	32360327	G	G/A	G	46	53	Sanger agrees with reference sequence
Intergenic	32559516	C	T	T	53	57	Sanger sequencing agrees with NGS (ref seq errors)
Intergenic	32705528	C	C/T	C	119	53	Sanger agrees with reference sequence
Intergenic	32714960	C	C/A	C	76	53	Sanger agrees with reference sequence
Intergenic	32753753	T	T/G	T	137	53	Sanger agrees with reference sequence
Intron	33039755	T	T/G	T	84	53	Sanger agrees with reference sequence
Intron	33081115	T	T/G	T	221	53	Sanger agrees with reference sequence
Intron	33134364	T	T/G	T	457	53	Sanger agrees with reference sequence
Intron	33147589	T	T/G	T/G	227	57	Sanger sequencing agrees with NGS
Intron	33171247	A	A/C	A	101	53	Sanger agrees with reference sequence
Intron	33234563	A	A/G	A	180	53	Sanger agrees with reference sequence
Exon	33257396	A	A/T	A	98	53	Sanger agrees with reference sequence

Exon	33269394	G	G/A	G	76	53	Sanger agrees with reference sequence
Intron	33280795	G	G/T	G	54	53	Sanger agrees with reference sequence
Intron	33289931	T	T/G	T	182	53	Sanger agrees with reference sequence
Intron	33383344	G	G/A	G	554	57	Sanger agrees with reference sequence
Intergenic	33457013	T	A	A	94	53	Sanger sequencing agrees with NGS (ref seq errors)
Intergenic	33457926	T	C	C	58	53	Sanger sequencing agrees with NGS (ref seq errors)
Intergenic	33481405	T	T/C	T	40	53	Sanger agrees with reference sequence
Intergenic	33505847	A	A/G	A	81	53	Sanger agrees with reference sequence
Intron	33556608	A	A/G	A	44	53	Sanger agrees with reference sequence
Intergenic	33563140	T	T/G	T	318	53	Sanger agrees with reference sequence
Intergenic	33583039	G	A	A	82	57	Sanger sequencing agrees with NGS (ref seq errors)
Intergenic	33583113	C	A	A	71	57	Sanger sequencing agrees with NGS (ref seq errors)
Intergenic	33584003	T	C	C	91	57	Sanger sequencing agrees with NGS (ref seq errors)
Intergenic	33586057	G	C	C	74	57	Sanger sequencing agrees with NGS (ref seq errors)
Intergenic	33586748	T	C	C	67	57	Sanger sequencing agrees with NGS (ref seq errors)
Intergenic	33587065	A	G	G	107	57	Sanger sequencing agrees with NGS (ref seq errors)
Intergenic	33587075	T	C	C	104	57	Sanger sequencing agrees with NGS (ref seq errors)
Intergenic	33587322	T	A	A	93	57	Sanger sequencing agrees with NGS (ref seq errors)
Intergenic	33587824	C	G	G	116	57	Sanger sequencing agrees with NGS (ref seq errors)
Intron	33590061	C	T	T	57	57	Sanger sequencing agrees with NGS (ref seq errors)
Intron	33590291	C	T	T	46	57	Sanger sequencing agrees with NGS (ref seq errors)
Intron	33590471	T	C	C	83	57	Sanger sequencing agrees with NGS (ref seq errors)
Intron	33592848	C	G	G	46	57	Sanger sequencing agrees with NGS (ref seq errors)
Intron	33592881	A	C	C	40	57	Sanger sequencing agrees with NGS (ref seq errors)
Intron	33593577	C	A	A	64	57	Sanger sequencing agrees with NGS (ref seq errors)
Intron	33595525	T	C	C	95	57	Sanger sequencing agrees with NGS (ref seq errors)
Intron	33595599	T	C	C	76	57	Sanger sequencing agrees with NGS (ref seq errors)
Intron	33596499	C	G	G	56	57	Sanger sequencing agrees with NGS (ref seq errors)
Intron	33596868	C	T	T	83	57	Sanger sequencing agrees with NGS (ref seq errors)
Intron	33598456	C	G	G	42	57	Sanger sequencing agrees with NGS (ref seq errors)
Intron	33598613	T	C	C	50	57	Sanger sequencing agrees with NGS (ref seq errors)
Intron	33598642	G	A	A	46	57	Sanger sequencing agrees with NGS (ref seq errors)
Intron	33600895	C	A	A	32	57	Sanger sequencing agrees with NGS (ref seq errors)
Intron	33607224	G	A	A	173	57	Sanger sequencing agrees with NGS (ref seq errors)
Intron	33608379	T	C	C	177	57	Sanger sequencing agrees with NGS (ref seq errors)
Intron	33609965	C	T	T	86	57	Sanger sequencing agrees with NGS (ref seq errors)
Intron	33610350	A	G	G	163	57	Sanger sequencing agrees with NGS (ref seq errors)
Intron	33610705	A	G	G	75	57	Sanger sequencing agrees with NGS (ref seq errors)
Intron	33617403	T	C	C	65	57	Sanger sequencing agrees with NGS (ref seq errors)
Intron	33617464	C	T	T	59	57	Sanger sequencing agrees with NGS (ref seq errors)
Intron	33618162	A	T	T	41	57	Sanger sequencing agrees with NGS (ref seq errors)

Supplemental Table 4. Results of Sanger validation of high-confidence variants.

	Capture Oligonucleotide Sequence (Primers)
**EGFR**	AGGGATGGGTCACAATGTTGAGGAC
	CAACCAAGCATTGAAGGAGCAGGTC
	CAGGGCCATCACCACACAAAGTGAAG
	CCTGGTGACCAAGCACATCAGA
	CCTGAGTGGAAGAAAGGTGGTTTAAC
	GAACACAGAGAAAGCCACCACCACG
	CTTGGACGCTGCCAGGTGACTCTAA
	GAGAGAAAGCCGTGGGGACTACAGA
	GGCTGTGCTCTGCCATCGTACAAAT
	TACAGACAGCTCCTCCACAAGGTGC
**KCNJ2**	TGGGTGACCTTCACACTGTACCTC
	CAGTGTCCTTGAGGTGCAGTGAGAC
	ACTTACTGACGTGGAGCAACGTG
	GGTGACACCTCAGATAAGTCTAAACCT
	CGAAAGATTGCAACTTTGGCTGCTA
	GTGGGTTGATGCTGGAGCTAAATAG
	GTGGCAGTGATGAGTCTGTGCATAA
	GATATTCATGGTGAGTCCCCTCCGT
	GGGACAGTGGTTAAAACAATGAGAGG
	GTAGCAAGATTCAGGCAACTGGTAA
**BRCA2**	GTGAGTAAACAGGTGGATGTGTGAG
	TCGGTCAGATACTGACGGTTGGGAT
	AGTATGCCTTTAGGCTGTAGTGTTGTC
	CAGCTAATCACATGACTGCTCAATG
	GCTAATAGATGCCTAAGCCCAGAAAG
	CGCCAGTAGGCTTGCTCAACATAGT
	GACTCTGAGCCTTCCTGTGCTTAGT
	CGGTTTGGAAACTCTGTGTAGGAAT
	CAGTCTGTCCCACCATGCTAAATTC
	CTGCTTAGTCCTGCCTCATTCAAGA
	GACAAAGGTGAGAGAGCATCCAGAT
**CLEC16A**	GGACAAGTCGCTTAATTTCTCAGA
	CTTGTCTCCGTCTGGGTTGG
	TGAATTGATTTACCCCGCCTG
	CACAGATATGCAACCTAAGGCCC
	TGTGTTCATGGGGTAAAATGGC
	AGCTCCAAAGTGCCAGGAGC
	CACAGGAATTGGTTACTCAGGCA
	GACAGCAGTTGCCAAGAGGG
	CAACTTCTCAAGAGTCTGAGCTGC
	CCAGCTGTGTGAAAGCAGAACC
	AATGAGTGGTGTGTAAAGGCAGCCGGA
	TTTGGGGTACTCTGTGCCTCC
	CCCAACAAATCTCTGGAAAACC
	TGAATATGTTGCTGCCCGTTTGACACC
	TACGACTCATGGCCCCACAC
	GGTAACCCTAGGCCCTGCTG

Supplemental Table 5. 46 RSE primers designed to capture the four genes (~700kb in total) in order to examine the effect of RSE primer spacing on capture efficiency (Figure 3).

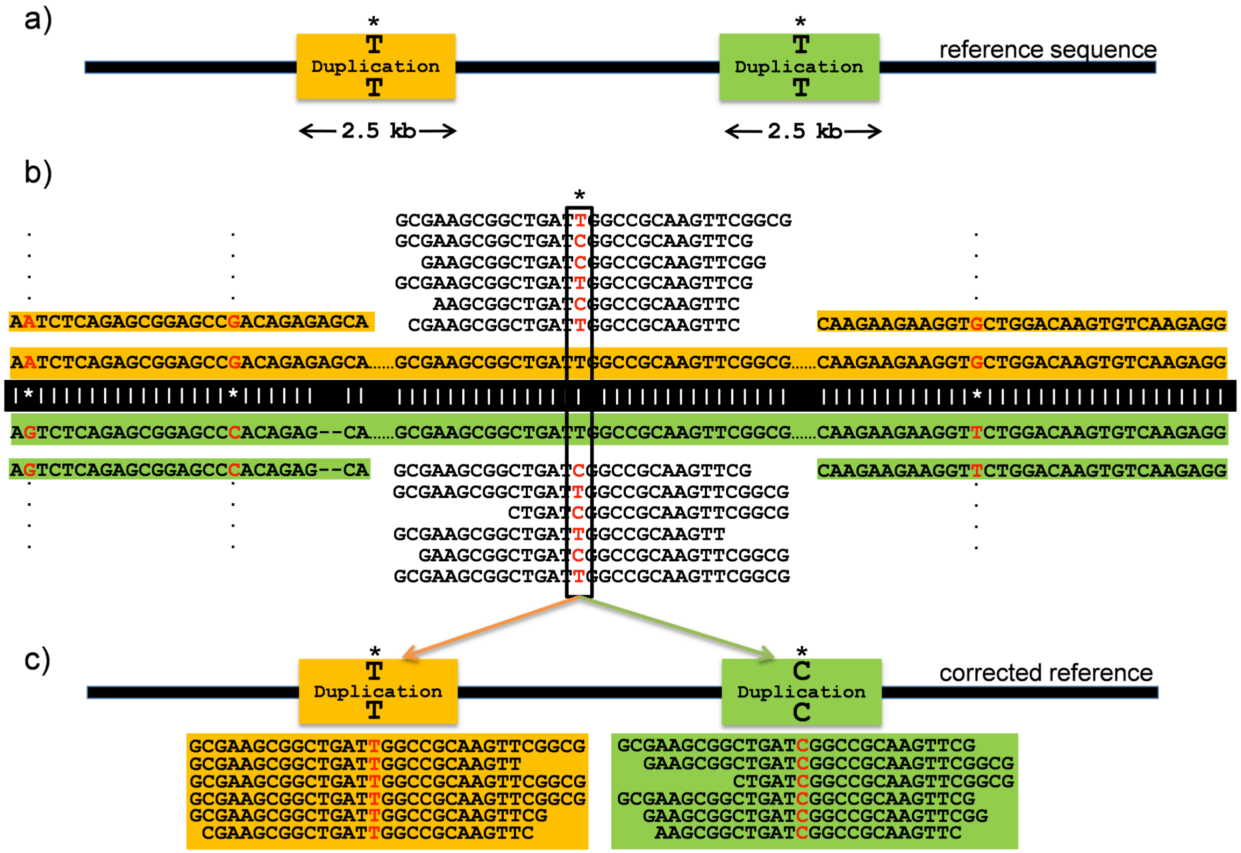

Supplemental Figure 1. Read segregation problems caused by reference sequence error in regional duplication. A 2.5kb duplication (a) within the MHC presented difficulties during the alignment of reads to this region. Reads at the beginning and ends of the duplicated regions segregated cleanly due to polymorphic differences between the duplications. But reads aligning to the middle of the duplication identified a variant position (marked by an *) (b). Sanger sequencing of this region identified that the reference sequence incorrectly identified the * base in the second duplication as a T, when it was actually a C, creating a false variant position. After correcting the reference sequence (c), the central reads segregated correctly and the false variant was eliminated.
